# Effects of vasectomy on breeding-related movement and activity in free-ranging white-tailed deer

**DOI:** 10.1186/s40462-025-00554-5

**Published:** 2025-05-14

**Authors:** Vickie DeNicola, Stefano Mezzini, Petar Bursać, Pranav Minasandra, Francesca Cagnacci

**Affiliations:** 1 White Buffalo Inc., 26 Davison Road, East Haddam, CT 06469 USA; 2 Present Address: Field Engine Wildlife Research and Management, 26 Davison Road, East Haddam, CT 06469 USA; 3 Animal Ecology Unit, Research and Innovation Centre, Fondazione Edmund Mach, San Michele all’Adige, TN Italy; 4 Center for Agriculture, Food and Environment, University of Trento, Trento, TN Italy; 5 Department of Biology, University of British Columbia Okanagan, Kelowna, BC Canada; 6 Okanagan Institute for Biodiversity, Resilience, and Ecosystem Services, The University of British Columbia Okanagan, Kelowna, BC Canada; 7 Department of Geodesy and Geoinformatics, Faculty of Civil Engineering, University of Belgrade, Belgrade, Serbia; 8 Department for the Ecology of Animal Societies, Max Planck Institute of Animal Behavior, Konstanz, Germany; 9 Department of Biology, University of Konstanz, Konstanz, Germany; 10 International Max Planck Research School for Organismal Biology, Konstanz, Germany; 11 Centre for the Advanced Study of Collective Behaviour, University of Konstanz, Konstanz, Germany; 12 National Biodiversity Future Center, Palermo, Italy

**Keywords:** Accelerometer, Contraception, Ctmm, Fertility control, GPS, *Odocoileus* Sp, Population management, Suburban wildlife

## Abstract

**Background:**

An abundance of white-tailed deer (*Odocoileus virginianus*) in suburban communities can lead to problems such as increased deer-vehicle collisions (DVCs), tick-borne illnesses, and forest degradation. Deer populations can be managed using traditional lethal methods; however, these methods are often impractical, ineffective, or socially unacceptable, prompting interest in management alternatives, including fertility control. Some fertility control methods (such as vasectomy, tubal ligation, and porcine zona pellucida-based vaccines) cause unsuccessfully bred females to experience multiple estrous cycles, potentially altering their movement behavior and fine-scale activity. Such changes could increase the risk of DVCs and negatively affect the physical condition of the animals. However, the effects of such treatments on animal behavior remain poorly understood, specifically in terms of breeding-related movements and energetics. This study aimed to evaluate the behavioral impacts of a large-scale vasectomy program on white-tailed deer.

**Methods:**

We conducted a 2-year study using a treatment/control design and analyzed biologging data of white-tailed deer at two sites near New York City, USA. We used a moving-window approach to assess the effects of a large-scale vasectomy program on the seasonal changes in movement behavior (home-range size, distance traveled, diffusion, and excursivity) and fine-scale activity (time spent in low-activity states and the daily number of state transitions).

**Results:**

There were no biologically significant differences in movement behavior or activity trends in either sex between the treatment and control groups. Females in both groups exhibited similar trends in all movement metrics, but females at the treatment site tended to switch between activity states more often in winter. Males at the treatment site expanded their space use less than control males during peak breeding season but otherwise exhibited similar movement behavior trends. Mortality rates and causes were similar at both sites.

**Conclusions:**

The vasectomy program, despite causing extra estrus periods in unsuccessfully bred females, is unlikely to cause appreciable behavioral changes that could exacerbate management-related issues at the time scales investigated. Fertility control methods inducing extra estrus periods could be implemented alone or alongside other strategies to reduce abundant deer populations with minimal impact on behavior.

**Supplementary Information:**

The online version contains supplementary material available at 10.1186/s40462-025-00554-5.

## Background

Over the last century, deer populations have expanded in suburban and urban areas across North America, Europe, and Asia, leading to a range of human-deer conflicts and ecosystem impacts [1]. Among the most pressing concerns are deer-vehicle collisions (DVCs) [2], damage to agricultural and residential landscapes [3], overbrowsing of native vegetation [4, 5], and the spread of tick-borne diseases [6]. Local reductions in deer densities, achieved through lethal methods (e.g., controlled hunting and culling), translocation, and fertility control, have been shown to reduce these conflicts [5, 7, 8]. While lethal methods remain the most common approach, they are often impractical, ineffective, or socially unacceptable in densely populated suburban and urban environments, leading to increased interest in non-lethal alternatives like fertility control [9].

Fertility control methods include surgical (e.g., vasectomy, ovariectomy, and tubal ligation) and non-surgical (e.g., immunocontraceptive vaccines and hormonal methods) options [10]. These approaches aim to reduce or eliminate reproductive output, and over time, lower population densities. These techniques have shown success in cases where reproduction is reduced to near zero, and immigration is minimal [11, 12]. However, some fertility control techniques prevent conception without halting estrous cycles in unbred females, leading to concerns that this behavioral change could alter movement patterns and increase human-wildlife conflicts. In particular, treatments such as vasectomy, tubal ligation, and porcine zona pellucida (PZP) immunocontraceptive vaccines result in repeated estrous cycling in unsuccessfully bred females, which may prolong movement-intensive breeding behaviors and elevate the risk of DVCs [13–15]. Several studies suggest that DVC rates increase during the breeding season due to increased movement and activity [16, 17]. Concerns about movement change due to repeated estrous cycling are frequently cited in debates over the suitability of fertility control as a management tool but have rarely been tested in free-ranging populations [10, 13–15].

Breeding-related movement behavior in male and female white-tailed deer (*Odocoileus virginianus* Zimmerman; hereafter, deer) has been studied extensively [18–22]. The breeding season results in the first annual estrus period in females and peak testosterone levels in males [23, 24]. Female deer generally show increased movement and activity during the breeding season, with peak activity occurring around conception [22]. Excursive behavior may increase a female’s chances of encountering a male during receptivity, especially in populations with low densities or female-biased sex ratios [18, 22]. Male deer exhibit distinct movement patterns during the breeding season, characterized by increased activity and larger home ranges [20, 21]. Females who fail to conceive due to fertility control measures may have repeated estrous cycles (∼ 1–2 days every ∼ 25 days), potentially into late winter [13–15]. These additional cycles may lead to extended breeding activity and movement [14, 15], which, in turn, may increase the animals’ energetic expenditures and the risk of DVCs [16, 17, 25]. Evaluating the overall effectiveness and applicability of fertility control treatments thus requires understanding the treatment’s side effects on animal behavior [10]. This research aimed to address these concerns by quantitatively assessing the implications of a large-scale vasectomy program that induces multi-estrous cycling in female deer through a semi-experimental, multi-scale, treatment-control study.

Between 2016 and 2024, New York City Parks and Recreation conducted an in-situ research program at Staten Island, New York, USA, to assess the potential effectiveness of a large-scale male vasectomy program in reducing the local deer population [26]. This study aimed to assess the behavioral impacts of this fertility control initiative, providing critical insights into its effects on movement behavior and activity in an urban/suburban deer population. Between 2021 and 2023, we collected Global Positioning System (GPS) and accelerometry data from biologging devices on male and female deer at the treatment site and compared them with the data of untreated males and females at a nearby control site. We examined the movement and activity patterns over time, focusing on trends during the expected period of additional estrous cycles (January–April; see Additional file 1: Table S1). We hypothesized that females at both sites would exhibit increased movement and activity during estrus periods, but that male movement and activity would peak only during the November–December breeding season, regardless of treatment status due to naturally declining testosterone levels [23]. By quantifying these patterns, this study provides much-needed empirical evidence on the behavioral impacts of fertility control, informing future management decisions in suburban deer populations.

## Methods

### Study areas

#### Treatment site—Staten Island, New York, USA

**Fig. 1:**
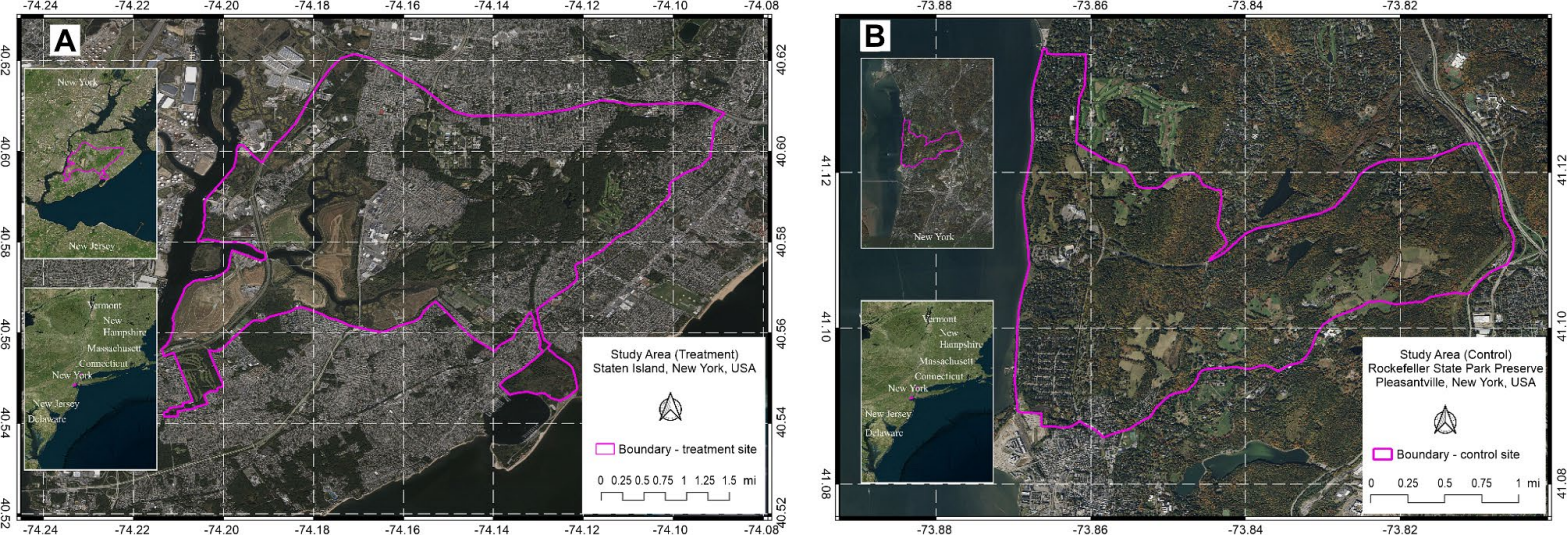
Maps of treatment and control study areas. **A** Map of the treatment study area in Staten Island and its location in New York State and the northeastern United States. **B** Map of the control study area in Rockefeller State Park Preserve in the context of New York State and the northeastern United States. The control site is located ∼ 50 km north of the treatment site

Staten Island (SI) is a borough of New York City (NYC), New York, USA (40.598237, − 74.144319; Fig. 1A) that covers an area of ∼ 155 km^2^, ∼ 35% of which is considered deer habitat (e.g., grassland, deciduous hardwood forest, and marsh areas greater than ∼ 2 ha) [26]. It is the least populated of the five boroughs of NYC (2022 human population: 495,925) and contains nearly 5,000 ha of protected parkland. The remaining area is a mix of single- and multi-family housing development, commercial development, other open spaces (e.g., golf courses and cemeteries), and roads. Over 97% of antlered SI males have been vasectomized, and hunting is prohibited within NYC limits [26]. We focused our capture efforts on The Greenbelt and Freshkills areas of SI (bounded area in Fig. 1A), specifically targeting these areas to closely resemble the contiguous open space at the control site. The area used by the individuals in this study encompassed 45.7 km^2^ of open spaces and developed areas.

#### Control site—Rockefeller State Park Preserve, Pleasantville, New York, USA

The Rockefeller State Park Preserve (RSPP; 41.107083, − 73.839131; Fig. 1B) is located in Pleasantville, New York, USA, in Westchester County. This site is ∼ 50 km north of the treatment site. The preserve encompasses 7.2 km^2^ of deciduous hardwood forests, agricultural pastures, grasslands, and aquatic habitats (J. DiPaola; personal communication; 13 February 2023). The forested area is dominated by several species of oak (*Quercus* spp.), tulip poplar (*Liriodendron tulipifera*), maple (*Acer* spp.), and American beech (*Fagus grandifolia*). Several water bodies are in the preserve, including the centrally located 7.7 ha Swan Lake and numerous interconnected streams. An ∼ 88.5 km network of carriage roads serves as trails for both pedestrian and equestrian traffic. Several roadways run adjacent to and intersect portions of the preserve, with certain boundaries of the park demarked by fences 2.5 m or higher. Many of these fences lack consistent maintenance and feature breaches that facilitate wildlife passage. The preserve is surrounded by ∼ 8.1 km^2^ of residential property with lot sizes varying from 0.4 to 5 ha. During the first year (2021–2022), deer hunting was allowed in the eastern section of the preserve (east of Route 448). Between September and December 2022, several hunters were also active in small areas on the northwestern side of the park. None of the study deer and only two non-study animals were harvested during the study period. We focused our capture efforts in areas where hunting was restricted. The area used by the individual deer in this study comprised 14.1 km^2^ of open spaces and developed areas.

At both study sites, peak breeding normally occurred in mid-November, with the breeding season starting in late October and ending in late December [23].

#### Capture and data collection

We aimed to collect the data from a minimum of 10 adult males and 20 adult females at each study site during each of the two study years, and we captured additional individuals when mortalities occurred. We immobilized adult deer using dart projectors [27] and the capture protocol outlined in DeNicola & DeNicola [12]. All captured deer were fitted with biologging devices (Vertex Plus with a 32 Hz Advanced 3-axis acceleration sensor; Vectronic Aerospace GmbH, Berlin, Germany) that contained GPS loggers, triaxial accelerometers, and an automatic drop-off mechanism. We estimated the weight based on chest girth measurements and age based on tooth wear [28]. No fawns or yearlings (deer younger than two years old) were handled to better reflect the expected age structure in a high-treatment vasectomy program, where the population would primarily consist of adult individuals if reproduction were reduced or eliminated. While approximate ages were estimated, all individuals were classified as adults due to the inherent imprecision of live aging in deer. All capture and handling procedures were conducted in accordance with the New York Department of Environmental Conservation Scientific Collection Permit (LCP #2100). All male deer included in the study at the treatment site underwent vasectomies during the previous field seasons, and ∼ 97% of antlered male deer at the treatment site were vasectomized [26]. Population dynamics are beyond the scope of this study.

We scheduled the GPS devices to obtain 1-h fixes. To extend the battery life, only six daily GPS fixes (one every 4-h) were transmitted via satellites. The remaining 18 daily fixes were stored in the collar until retrieval. Triaxial accelerometers were used to record data at 32 Hz with a sensitivity of ±4 g (surge (X-axis), sway (Y-axis), and heave (Z-axis)). We defined the behavioral periods as pre-breeding (22 September–18 October), breeding (20 October–4 January), peak breeding (10 November–30 November), and post-breeding/extra estrus periods (5 January–1 April) [23]. During Year 1 (August–May 2022), collars were removed on 30 May 2022 to ensure device refurbishment for Year 2 deployment. In Year 2 (August 2022 to June 2023), the collars were removed on 20 June 2023. Data were collected between the device deployment and remote drop-off or animal death.

#### GPS data analyses

We preprocessed the raw GPS data after downloading from MoveBank (MoveBank Study ID: 1879977576; Additional file 5: Section S1) and then estimated the changes in movement over time for each animal using the continuous-time speed and distance (CTSD) method [29]. This method is based on the continuous-time movement modeling workflow via the ctmm package (1.2.0) [30] in R (4.4.0) [31]. To quantify the changes in movement behavior, we modeled each animal’s telemetry data using a moving-window approach [32] (also see the empirical example by [33], in which each window had a size and slide of seven and three days, respectively, resulting in overlapping 7-day estimates. We selected a 7-day window to identify fine-scale temporal variations in movement behavior (e.g., peak breeding season, proestrus/estrus) [14, 23] while maintaining sufficiently abundant range crossings (e.g., frequent movements across the boundaries of the estimated home range to provide enough data points to define home range reliably) to produce reasonable estimates of home-range (HR) size [34]. Because deer collared after late August were not tracked during summer (Additional file 5: Figure S1B), the dates for each window were converted to the number of days after 1 August to ensure continuity between December and January of the following year (Additional file 5: Figure S1C).

We fit a movement model to each 7-day subset of telemetry data using the ctmm.select() function to determine the best-fitting model as a function of sampling frequency and the degree of autocorrelation in the tracking data [30]. From each model, we extracted the animal’s 7-day HR size and, when possible, the average daily anomalous diffusion, average daily distance traveled (i.e., average speed), and excursivity. Diffusion was calculated as the asymptote of the time-dependent expected square displacement over a finite period of time, which results in superdiffusion for time lags below an animal’s foraging timescale, linear diffusion for time lags greater than the foraging timescale but smaller than the range-crossing timescale, and asymptotic diffusion for time periods longer than the range-crossing timescale [35]. Excursivity was determined by calculating the daily mean quantile of each animal’s utilization distribution [36], which we estimated using Autocorrelated Kernel Density Estimation [37] on the full telemetry dataset of each individual (keeping the two years separate; see Additional file 6: Figure S1).

#### Modeling movement behavior

We estimated the differences in movement behavior over time between sexes at each site using the mgcv package [38] in R software to fit four hierarchical generalized additive models for location and scale (HGAMLS) [38–41]. Specifically, we fit HGAMLSs with gamma location-scale families of distributions to 7-day HR size, average daily distance traveled, and average daily diffusion (since all three were strictly positive; see Additional file 2: Table S1), whereas we modeled excursivity using an HGAMLS with a beta location-scale family of distributions. The code for the family was provided to us by Dr. Simon Wood, the developer and maintainer of the mgcv package, and is available in our GitHub repository.

All four models had the same set of three terms for both the mean and scale linear predictors: (1) A group-level fixed-effect intercept and smooth term of time (days since 1 August) accounted for differences across each combination of sex and treatment level. (2) A “sum-to-zero” smooth term of time between the two study years (for each combination of sex and treatment level) accounted for the differences between study years while weighing each year equally. (3) A factor-smooth interaction term for each animal in each study year accounted for individual-level deviations from the group-level mean. Additional details on the models’ structures are provided in Additional file 2.

#### Accelerometer data analyses

To quantify fine-scale movement behavior, we defined accelerometer-based activity states for each animal that we derived using vectorial dynamic body acceleration (VeDBA) [42], a rotationally invariant metric proportional to body movement that is strongly and positively associated with energy expenditure [43]. We estimated the static acceleration using the three mean acceleration axes (X, Y, and Z) in non-overlapping 2-s intervals, and we calculated the log mean VeDBA for each interval to quantify the intensity of activity [44]. To account for differences among devices, animals, and the positioning of the devices on animals’ necks, we defined behavioral states for each individual based on each collar’s local minima in the distributions of log mean VeDBA, with VeDBA = 0 (log mean VeDBA = − Inf) as a no-activity state. We thus had four states, namely no, low, medium, and high activity. We visually inspected the log mean VeDBA histograms for each month for 10 individuals to ensure consistent minima values over time. To quantify changes in daily behavior, we computed the daily proportion of time spent in each activity state and the daily number of transitions between different states. Additional details can be found in Additional file 7.

#### Modeling time in activity states and transitions

To estimate the effects of the treatment on fine-scale movement behavior, we used the mgcv R package [38] to fit hierarchical generalized additive models (HGAMs) to the proportion of time spent in the no- or low-activity states and the daily number of transitions between states (Additional file 2: Table S2). The first HGAM used a beta distribution and logit link function, whereas the second HGAM used a negative binomial family of distributions and a log link function. The HGAMs had the same terms as the HGAMLSs described in the previous section (Additional file 2: Table S1) but assumed a common and constant mean-variance relationship (i.e., a common and constant scale parameter).

## Results

### Capture and collaring

Between 23 August and 13 December 2021 (Year 1) and 14 August 2022 and 8 January 2023 (Year 2), 158 adult deer (a total of 52 males and 106 females in SI and RSPP; Table 1) were captured and fitted with biologging devices. Seven devices failed to transmit GPS fixes (Additional file 5: Figure S1A); therefore, we included only collar-stored GPS fixes (e.g., one fix/4-h) and accelerometry data for these animals in the analyses. In Year 1, we excluded 10 individuals from the study who died before the first frost due to unknown causes during a known epizootic hemorrhagic disease outbreak (the tracking duration was typically less than 45 days and not during the periods of interest). Similarly, we excluded 23 individuals from the study with tracking periods of < 28 days and one owing to collar failure. At both sites, eight females and two males were tracked in both years. Our final sample, therefore, included data from 21 males and 42 females in Year 1 and 19 males and 42 females in Year 2 (Table 1).

**Table 1:** Number of individuals captured and number of individuals included in the study (# individuals captured; # individuals included in the study) at the treatment site in Staten Island, NY, USA and at the control site in Rockefeller State Park Preserve, Pleasantville, NY, USA in both study years (2021–2022; 2022–2023)

	Year 1		Year 2	
	SI	RSPP	SI	RSPP
Females	34; 21	27; 21	24; 22	21; 20
Males	17; 11	13; 10	12; 10	10; 9

Among the animals in the study from Year 1, we recorded nine mortalities (collars collected on 30 May 2022), whereas in Year 2, we recorded five (collars collected on 20 June 2023; Additional file 3: Table S1). None of the animals in the study were harvested through hunting at the control site; however, poachers killed two study individuals at the control site. In the final set of monitored deer, we recorded seven DVC-related mortalities (4 in SI and 3 in RSPP) and four owing to unknown causes (1 in SI and 3 in RSPP). The mortality rate of individuals in the study was overall lower at the treatment site, but there were no significant differences at the α = 0.10 level, even within sexes. Fisher’s exact test produced the following estimated odds ratios (treatment relative to control): 0.55 overall (*P* = 0.3861), 1.4 (*P* = 1.0) for males, 0.29 for females (*P* = 0.1511).

### Movement behavior

We produced HR size estimates for all 7-day windows for all individuals and removed 99 estimates (1.05% of the total; Additional file 4: Table S1) because of a lack of range residency that resulted in excessively large estimates (> 10 km^2^; range: 10–179 km^2^). Only 60.3% of the ctmm movement models had sufficiently fine telemetry data to estimate the daily distance traveled, but almost all movement models (98.9%; Additional file 4: Table S1) could produce daily diffusion estimates.

**Fig. 2:**
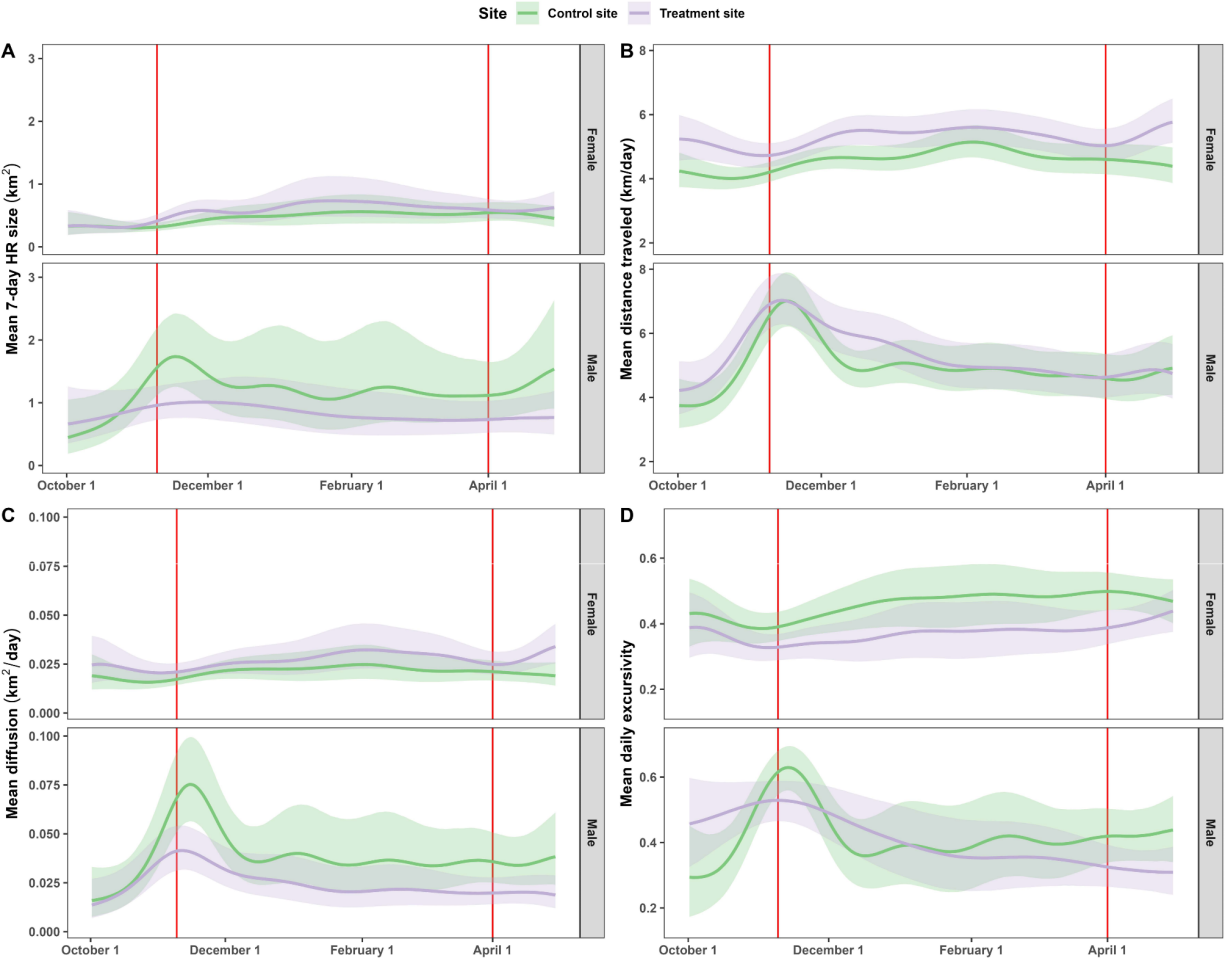
Trends in 7-day home range, daily distance traveled, daily diffusion, and daily excursivity. Trends in the mean 7-day home range (**A**), daily distance traveled (**B**), daily diffusion (**C**), and daily excursivity (**D**) over the day of the year by sex and treatment status. The 95% Bayesian credible intervals were estimated using a Gaussian assumption of the residuals and account for uncertainty in the scale parameter. The first red line indicates the beginning of peak breeding season (10 November), and the second indicates the end of the potential extra estrus periods (1 April)

Females in both groups exhibited similar sizes and trends over time in all four movement metrics (Fig. 2; Additional file 4: Tables S2 and S3). Males in the control site increased their 7-day HR size, daily distance traveled, daily diffusion, and daily excursivity around the peak of the November breeding season (all four approximate *p*-values < 2.7e-06; see Fig. 2, Additional file 4: Table S2 and Table S3). After early December, the daily distance traveled, daily diffusion, and excursivity measures declined to previous values and remained approximately constant, whereas the 7-day HR size exhibited a less noticeable increase and decrease. Males in the treatment site did not change their HR size significantly over time (approximate *p*-value = 0.60) and had less pronounced peaks in diffusion (approximate *p*-value: < 2e-16) and excursivity (approximate *p*-value: 7.87e-05), but we found no appreciable differences between the temporal trends in the mean daily distances traveled by the treated and control males. All four models explained ≥80% of the deviance.

### Activity

The log mean VeDBA distribution included three distinct peaks across all individuals in our study (Additional file 7: Figure S1). Upon inspecting the subset of 10 individuals, we found no differences in the histogram’s monthly local minima values compared with that of the full year (Additional file 7: Figure S2), indicating that these peaks could be used as activity levels to consistently characterize behavior across individuals.

**Fig. 3:**
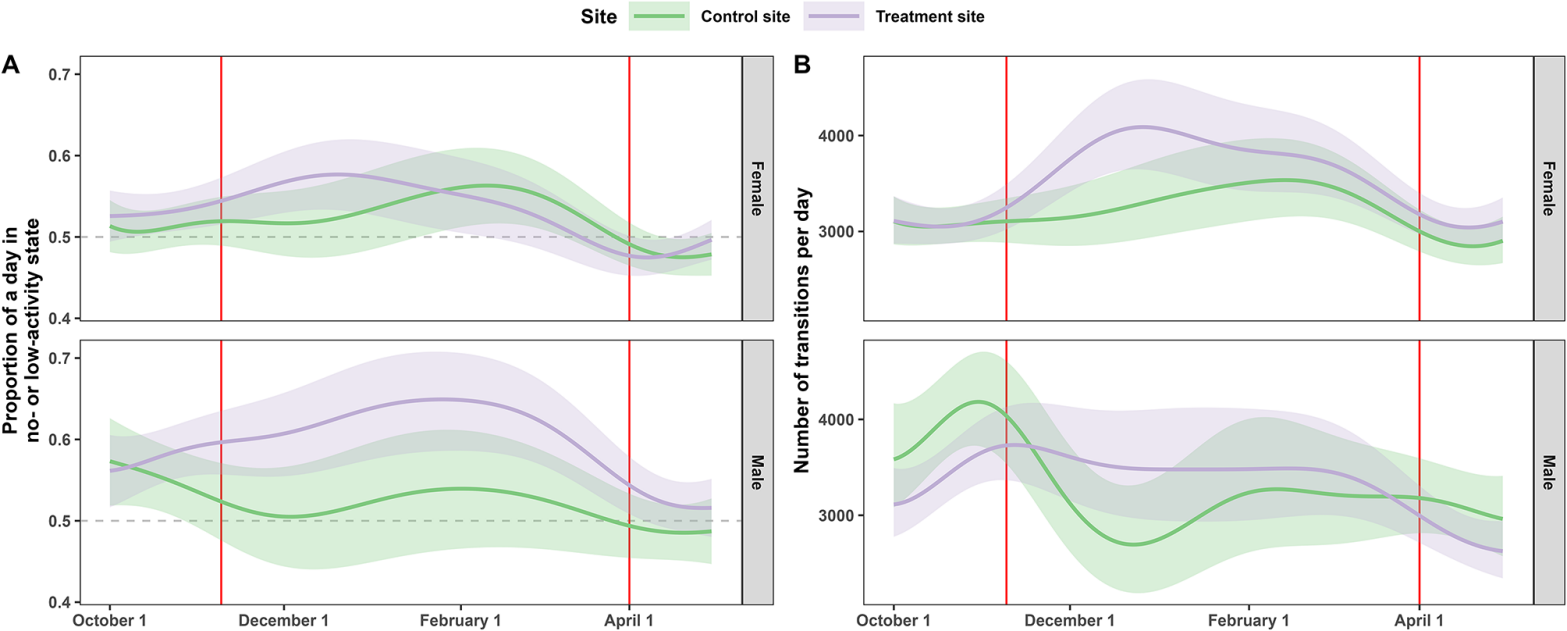
Trends in behavioral states and daily number of transitions. Mean trends over the day of the year in the proportion of time spent in the no- or low-activity state (**A**) and the daily number of transitions between states (**B**) by sex and treatment status. The colored line shows the model-predicted mean, whereas the shaded ribbons indicate the associated 95% Bayesian credible intervals (under the assumption of Gaussian residuals). The first red line indicates the beginning of peak breeding season (10 November), and the second indicates the end of the potential extra estrus periods (1 April)

Vasectomized males tended to spend a greater proportion of time in the no- or low-activity states, but we found no appreciable difference between females at the two sites (Fig. 3A; Additional file 4 Table S2 and Table S3). At both sites, males showed comparable increases in daily state transitions in fall and similar values throughout the year (Fig. 3B). Treatment-site females exhibited more daily behavioral transitions in winter, but there were no appreciable differences between fall, late winter, and spring (Fig. 3B). Both models explained most of the deviance (state: 64.4%, daily transitions: 70.7%).

## Discussion

This study examined the effects of a high-percentage vasectomy program (> 97%) on breeding-related movement and activity behaviors of free-ranging deer [26]. We focused on whether males and females in the treatment area exhibited prolonged breeding-related movements and increased activity beyond the peak breeding season due to repeated estrus periods in unsuccessfully bred females. We hypothesized that males would not exhibit any biologically significant differences in movement behavior or activity between sites, whereas females would show differences in fine-scale activity but not movement behavior during extra estrus periods (Additional file 1: Table S1). Overall, our findings revealed minimal differences in movement behavior and activity between the treatment and control sites, with no appreciable variations attributable to extra estrous cycling at the treatment site. Treatment site movement behavior and activity were consistent with the hormonally-driven patterns exhibited at the control site. At both sites, female patterns were relatively stable throughout the study period, and male movement behavior peaked in November and declined throughout winter. This consistency in pattern between sites supports the conclusion that fertility control methods leading to extra estrus periods can be implemented with minimal population-level behavioral alterations at the time scale studied.

### Female deer movement behavior and activity

As hypothesized, we did not find appreciable differences between the groups’ large-scale movement behaviors during the extra estrus periods. While this may be due to the brief duration of estrus bouts and pre/post conception activity (48–96 h) [14, 22] relative to the window size (7 days), narrower windows would have likely produced less accurate movement metrics (particularly HR size) owing to insufficient effective sample sizes [35]. A lack of synchronization across estrus periods could also cause the HGAMLS to fail to detect common oscillations, but sensitivity analyses showed little to no change in both the group-level and individual-level smooth terms, even for very large basis sizes (k = 30; not shown).

The slight tendency of treated females to spend more time in lower activity states while exhibiting more state transitions per day suggests that they may be less active overall but more responsive to nearby males during additional estrus periods. However, this difference does not appear substantial enough to negatively impact body condition, especially since females at the treatment site are unlikely to be gravid [26]. Existing studies comparing PZP-treated and untreated females indicate that non-gravid, non-lactating treated females tend to maintain or even improve body condition relative to gravid or lactating females [15]. This suggests that females at the treatment site may similarly maintain or improve body condition, as they are not subjected to the high metabolic demands of gestation, parturition, and lactation. Finally, mortality rates were lower at the treatment site, further suggesting that the program had minimal adverse effects and reinforcing the program’s safety.

### Male deer movement behavior and activity

Our analysis found no substantial differences in the movement behavior of male deer between treatment and control groups. While treated males had lower measures and smoother curves for HR size, diffusion, and excursivity during the breeding season, there were minimal variations thereafter, suggesting that both groups behaved similarly (e.g. males increase movement during peak breeding and decrease it in subsequent months). These patterns align with the findings of previous studies on breeding-related behavior [19–21] in which expanded breeding-related movement in male deer is linked to day length and peak testosterone levels [24]. As we expected, males at the treatment site did not maintain the larger breeding-related movement levels in January–March, despite female’s extra estrus periods. The flatter trends at the treatment site suggest a less pronounced peak breeding season. This could indicate that unsuccessfully bred females can enter estrus earlier in the year because they do not expend energy in gestation and lactation [45] and that males may remain active as unsuccessfully bred females return to estrus in mid-to-late December; however, their overall patterns remained similar to males at the control site. An alternative explanation for the less pronounced peak could be related to differences in habitat between the two sites. Suburban environments provide high-quality edge habitat, offering reliable anthropogenic food sources, such as ornamental plantings, bird feeders, and landscaped green spaces [46, 47]. These anthropogenic resources can reduce the need for long-distance foraging movements, potentially leading to more localized activity patterns compared to deer in more rural or agricultural settings [48–50]. Although the two sites had comparable habitat in areas where we captured deer, individuals on Staten Island ultimately occupied more habitat that included impervious surfaces (e.g., dwellings, office parks), which may have increased their access to anthropogenic resources and contributed to reduced overall space use (Additional file 8: Table S1) [49].

Contrary to previous observations on breeding behavior [20, 21], the 7-day HR size did not decrease to early-fall levels after December at either site. Testosterone-linked movements associated with breeding typically diminish in late December, leading to more localized, sedentary feeding behavior [24]. Nevertheless, there were clear differences in how males moved within their HR post-peak breeding (daily distance traveled, daily diffusion, and excursivity all declined), which suggests behavioral shifts following the breeding season.

Treatment-site males showed a marginally higher tendency to spend more time in the no- and low-activity states than control-site males, but the higher number of transitions between activity states, especially in December and January, may suggest extended responsiveness to females in estrus. However, this did not correlate with any variation in activity levels that could be associated with illness or compromised health [51, 52], such as an increased proportion of time in low-activity states indicating illness. This conclusion is also supported by the lack of unexplained male mortalities (see Methods) and a consistent long-term pattern in carcass pickups (no increase during or after extra estrus periods; Additional file 3: Table S2).

### Implications and future research

This study provides a comprehensive analysis of the breeding-related movement and activity patterns of free-ranging deer using precise, high-resolution data that surpasses the limitations of coarse datasets and captive animal studies that have influenced the existing literature. Our findings challenge the primary management concerns related to fertility control programs, specifically the potential for increased DVCs and reduced body condition due to prolonged breeding-related movements. We observed no evidence of negative outcomes during the study period, suggesting that vasectomy programs may induce negligible behavioral and social changes without significant impacts on population health or public safety. To further bolster our conclusions, closely monitored DVCs have declined by 75%, and the overall population has declined by 45% since the inception of the vasectomy program in 2016 [26]. However, the results of this study should not be assumed to be universally applicable to other ungulate populations. Nevertheless, the absence of appreciable negative outcomes in this context supports the continuous use of this program as a safe and humane management tool.

However, several limitations must be considered. Movement patterns may have been influenced by the fragmented suburban habitat at the treatment site and the agriculturally productive habitat at the control site [46, 47]. However, we were able to observe movement changes in males during the peak breeding season and subsequent declines post-breeding regardless of treatment status. We would expect to see this behavioral change in any habitat at any density. Additionally, we could not determine the reproductive status of all females, though we assumed most treatment-site females were non-reproductive based on population estimates and a ∼ 95% reduction in fawning at the treatment site [26]. Investigating the effects of late gestation and parturition on female movement could provide additional insights. Future research on the effects of age-class distribution and density on breeding-related movements would facilitate the evaluation of the broader implications of this program.

## Conclusions

We presented the first fine-scale study of the effects of a high-percentage vasectomy program on a population of deer. While fertility control methods are often disregarded owing to concerns about behavioral changes, we used a multi-scale, treatment-control, semi-experimental design with robust quantitative analysis to demonstrate that using high-percentage vasectomies as a means of population control had no biologically significant adverse effects on deer’s movement behavior or activity. The approach used in this study can inform broader management practices, and this framework may be adapted to evaluate other wildlife management interventions, offering valuable insights into their long-term ecological impacts.

## Electronic supplementary material

Below is the link to the electronic supplementary material.

Additional file 1: Table S1: Summary of the study design and related hypotheses, predictions, data, consequences, and methods used in assessing the impact of vasectomy on breeding-related movement behavior and activity in free-ranging white-tailed deer

Additional file 2: Table S1 and S2: Hierarchical generalized additive models for location and scale (HGAMLSs) used to estimate the effect of the vasectomy treatment on movement behavior and activity states

Additional file 3: Tables S1 and S2: Mortality data and cause for individuals included in the movement study; and individual deer carcasses (monthly percentage of total) that were reported to the New York Department of Sanitation for pick-up on both public and private lands

Additional file 4: Tables S1, S2, and S3: Percentage of movement parameter estimates used in the analysis after excluding NA, non-finite, and excessively large value, families of distributions; percentages of deviance explained, and scale estimates ($\:\widehat{\theta\:}$) for HGAMs; and model summaries for hierarchical generalized additive models for location and scale (HGAMLSs) and hierarchical generalized additive models (HGAMs)

Additional file 5: Section S1 and Figure S1: Detailed description of data cleaning and processing for telemetry data from both study sites and median GPS fix rate for individuals in the final sample

Additional file 6: Figure S1: Telemetry data (dots) and autocorrelated kernel density estimate (shading) for deer 148 in Year 1 (2021–22) with a two-point equidistant projection

Additional file 7: Figures S1 and S2: Monthly trimodal distribution of log mean VeDBA values for 2-s intervals for one individual and trimodal distribution of log mean VeDBA values for 2-s intervals for one individual. Low (log(VeDBA) < 1.12), medium (1.12 < log(VeDBA) < 4.2), and high (log(VeDBA) > 4.2) activity states are represented by the three distribution peaks

Additional file 8: Table S1: Percentage habitat composition for female and male deer at treatment and control sites in Year 1. Habitat composition was determined using GLC_FCS30D land cover data (Liangyun et al. 2023) overlaid with the 50% home range estimated via Autocorrelated Kernel Density Estimation (AKDE) in ctmm (Calabrese et al. 2016). The area of each habitat type within the 50% AKDE was calculated for each individual, and group-level percentages represent the habitat composition across all individuals in that category

## Data Availability

Data and code are available on GitHub at https://github.com/StefanoMezzini/ny-deer-vasectomy, where we provide all derived data required to run our analysis (e.g., R code and data). Raw GPS data supporting this research are sensitive and not available publicly but are available to qualified researchers; use of these data will be restricted to research, and users will not be allowed to distribute the data. Raw GPS data for white-tailed deer are owned by Field Engine Wildlife Research and Management and are available by contacting the organization (Vickie.denicola@fieldengine.com) and requesting the GPS data from New York City white-tailed deer vasectomy program between 2021 and 2023.

## Abbreviations

DVC: Deer-Vehicle Collision
GPS: Global Positioning System
SI: Staten Island
RSPP: Rockefeller State Park Preserve
HR: Home Range
