# Supplementary material for: Effects of vasectomy on breeding-related movement and activity in free-ranging white-tailed deer
Source: Mov Ecol. 2025 May 14;13:34. doi: 10.1186/s40462-025-00554-5 (PMC12079978; doi:10.1186/s40462-025-00554-5)
Supplement: Supplementary file 1 — Additional file 1: Table S1: Summary of the study design and related hypotheses, predictions, data, consequences, and methods used in assessing the impact of vasectomy on breeding-related movement behavior and activity in free-ranging white-tailed deer [file 40462_2025_554_MOESM1_ESM.docx]

# Additional file 1

**Effects of vasectomy on breeding-related movement and activity in free-ranging white-tailed deer**

Vickie DeNicola, Stefano Mezzini, Petar Bursać, Pranav Minasandra, and Francesca Cagnacci

### Table S1. Summary of the study design and related hypotheses, predictions, data, consequences, and methods used in assessing the impact of vasectomy on breeding-related movement behavior and activity in free-ranging white-tailed deer. This study was conducted at a treatment site in Staten Island, NY, USA (SI) and at a control site in Rockefeller State Park Preserve, Pleasantville, NY, USA (RSPP) between 2021 and 2023.

| Hypothesis | Prediction (Control-RSPP) | Prediction (Treatment- SI) | Data Source and Measure | Consequence | Method |
| --- | --- | --- | --- | --- | --- |
| Male white-tailed deer increase exploratory behavior in response to elevated testosterone levels. | Male white-tailed deer at the control site will show peak home range, distance traveled, diffusion, and excursivity during late October - December. These measures will decrease as testosterone levels drop in early January. | No expected differences with respect to Control | 1 GPS fix/hour; 7-day home range, daily distance traveled, daily diffusion, daily excursivity | Increased deer-vehicle collisions, decreased body condition | Measures derived via ctmm in R with 7-day moving window/3-day slide; Modeled with HGAMLS |
| Female white-tailed deer show increases in exploratory behavior in response to estrus periods. | Female white-tailed deer at the control site will show increases in home range, distance traveled, diffusion, and excursivity in November - December during a window around their estrus period. Owing to the short duration of estrus, these changes may not be noticeable in the modeled data. | No expected differences with respect to Control, despite extra-estrus, as these changes might not be noticeable in the modeled data. | 1 GPS fix/hour; 7-day home range, daily distance traveled, daily diffusion, daily excursivity | Increased deer-vehicle collisions, decreased body condition | Measures derived via ctmm in R with 7-day moving window/3-day slide; Modeled with HGAMLS |
| Male white-tailed deer become more active with elevated testosterone levels. | Male white-tailed deer at the control site will show peak activity during late October - December. These measures will decrease as testosterone levels drop in early January. | No expected differences with respect to Control | 32 Hz tri-axial accelerometry; # of activity state transitions/day; proportion of time/day spent in no/low activity state | Increased deer-vehicle collisions, decreased body condition | Derived measures using 2-s window log (VeDBA); states identified via clustering with GMM; modeled with HGAM |
| Female white-tailed deer show increases in activity in response to estrus periods. | Female white-tailed deer at the control site will increase activity in November - December during a window around their estrus period. Owing to the short duration of estrus, these changes may not be noticeable in the modeled data. | Female white-tailed deer at the treatment site will increase in activity from November - March during a window around their estrus periods (extra-estrus). | 32 Hz tri-axial accelerometry; # of activity state transitions/day; proportion of time/day spent in no/low activity state | Increased deer-vehicle collisions, decreased body condition | Derived measures using 2-s window of log (VeDBA); states identified via clustering with GMM; modeled with HGAM |
