# Supplementary material for: Effects of vasectomy on breeding-related movement and activity in free-ranging white-tailed deer
Source: Mov Ecol. 2025 May 14;13:34. doi: 10.1186/s40462-025-00554-5 (PMC12079978; doi:10.1186/s40462-025-00554-5)
Supplement: Supplementary file 2 — Additional file 2: Table S1 and S2: Hierarchical generalized additive models for location and scale (HGAMLSs) used to estimate the effect of the vasectomy treatment on movement behavior and activity states [file 40462_2025_554_MOESM2_ESM.docx]

# Additional file 2

**Effects of vasectomy on breeding-related movement and activity in free-ranging white-tailed deer**

Vickie DeNicola, Stefano Mezzini, Petar Bursać, Pranav Minasandra, and Francesca Cagnacci

### Table S1. Hierarchical generalized additive models for location and scale (HGAMLSs) used to estimate the effect of the vasectomy treatment on movement behavior. The response variables are 7-day 95% home range size (km^2^; hr_est_95), daily distance traveled (km/day; speed_est), daily diffusion (km^2^/day; diffusion_est), and daily excursivity (unitless; excursivity). The predictor variables are the four combinations of sex and treatment status (sex_treatment), day of year (days_since_aug_1), study year (study_year), and animal ID for each study year (i.e., each collaring event; animal_year).

| **Response** | **HGAMLS Model** |
| --- | --- |
| 7-day home range size (km^2^) | gam(formula = list(  # linear predictor for the mean  hr_est_95 ~  sex_treatment +  s(days_since_aug_1, by = sex_treatment, k = 15, bs = 'tp') +  s(days_since_aug_1, study_year, by = sex_treatment, k=15, bs='sz')+  s(days_since_aug_1, animal_year, k = 15, bs = 'fs',  xt = list(bs = 'cr')),  # linear predictor for the scale (sigma2 = mu^2 * scale)  ~ sex_treatment +  s(days_since_aug_1, by = sex_treatment, k = 15, bs = 'tp') +  s(days_since_aug_1, study_year, by = sex_treatment, k=15, bs='sz')+  s(days_since_aug_1, animal_year, k = 15, bs = 'fs',  xt = list(bs = 'cr'))),  family = gammals(),  data = d,  method = 'REML') |
| Distance traveled (km/day) | gam(formula = list(  # linear predictor for the mean  speed_est ~  sex_treatment +  s(days_since_aug_1, by = sex_treatment, k = 15, bs = 'tp') +  s(days_since_aug_1, study_year, by = sex_treatment, k=15, bs='sz')+  s(days_since_aug_1, animal_year, k = 15, bs = 'fs',  xt = list(bs = 'cr')),  # linear predictor for the scale (sigma2 = mu^2 * scale)  ~ sex_treatment +  s(days_since_aug_1, by = sex_treatment, k = 15, bs = 'tp') +  s(days_since_aug_1, study_year, by = sex_treatment, k=15, bs='sz')+  s(days_since_aug_1, animal_year, k = 15, bs = 'fs',  xt = list(bs = 'cr'))),  family = gammals(),  data = d,  method = 'REML') |
| Diffusion (km^2^/day) | gam(list(  # linear predictor for the mean  diffusion_est ~  sex_treatment +  s(days_since_aug_1, by = sex_treatment, k = 15, bs = 'tp') +  s(days_since_aug_1, study_year, by = sex_treatment, k=15, bs='sz')+  s(days_since_aug_1, animal_year, k = 15, bs = 'fs',  xt = list(bs = 'cr')),  # linear predictor for the scale (sigma2 = mu^2 * scale)  ~ sex_treatment +  s(days_since_aug_1, by = sex_treatment, k = 15, bs = 'tp') +  s(days_since_aug_1, study_year, by = sex_treatment, k=15, bs='sz')+  s(days_since_aug_1, animal_year, k = 15, bs = 'fs',  xt = list(bs = 'cr'))),  family = gammals(),  data = d,  method = 'REML') |
| Daily excursivity | gam(formula = list(  # linear predictor for the mean  excursivity ~  sex_treatment +  s(days_since_aug_1, by = sex_treatment, k = 15, bs = 'tp') +  s(days_since_aug_1, study_year, by = sex_treatment, k=15, bs='sz')+  s(days_since_aug_1, animal_year, k = 15, bs = 'fs',  xt = list(bs = 'cr')),  # linear predictor for the scale (sigma2 = mu * (1-mu) * scale)  ~ sex_treatment +  s(days_since_aug_1, by = sex_treatment, k = 15, bs = 'tp') +  s(days_since_aug_1, study_year, by = sex_treatment, k=15, bs='sz')+  s(days_since_aug_1, animal_year, k = 15, bs = 'fs',  xt = list(bs = 'cr'))),  family = betals(),  data = d,  method = 'REML') |

### Table S2. Hierarchical generalized additive models (HGAMs) used to estimate the effect of the vasectomy treatment on activity states. The response variables are the daily proportion of time spent in a no- or low-activity state (vs a medium- or high-activity state; p_low) and the daily number of transitions between activity states (no, low, medium, high; n_transitions). The predictor variables are sex and treatment status of individuals (sex_treatment), day of year (days_since_aug_1), study year (study_year), and animal ID in each study year (animal_year).

| Model | HGAM |
| --- | --- |
| Daily proportion of time  spent in no- or low-  activity state | bam(  p_low ~  sex_treatment +  s(days_since_aug_1, by = sex_treatment, k = 10, bs = 'tp') +  s(days_since_aug_1, study_year, by = sex_treatment, k=10, bs='sz')+  s(days_since_aug_1, animal_year, k = 10, bs = 'fs',  xt = list(bs = 'cr')),  family = betar(link = 'logit'),  data = d,  method = 'fREML',  discrete = TRUE) |
| Daily number of  transitions between  activity states | bam(  n_transitions ~  sex_treatment +  s(days_since_aug_1, by = sex_treatment, k = 10, bs = 'tp') +  s(days_since_aug_1, study_year, by = sex_treatment, k=10, bs='sz')+  s(days_since_aug_1, animal_year, k = 10, bs = 'fs',  xt = list(bs = 'cr')),  family = nb(link = 'log'), # data is clearly over-dispersed  data = d,  method = 'fREML',  discrete = TRUE) |
