# Supplementary material for: Effects of vasectomy on breeding-related movement and activity in free-ranging white-tailed deer
Source: Mov Ecol. 2025 May 14;13:34. doi: 10.1186/s40462-025-00554-5 (PMC12079978; doi:10.1186/s40462-025-00554-5)
Supplement: Supplementary file 3 — Additional file 3: Tables S1 and S2: Mortality data and cause for individuals included in the movement study; and individual deer carcasses (monthly percentage of total) that were reported to the New York Department of Sanitation for pick-up on both public and private lands [file 40462_2025_554_MOESM3_ESM.docx]

# Additional file 3

**Effects of vasectomy on breeding-related movement and activity in free-ranging white-tailed deer**

Vickie DeNicola, Stefano Mezzini, Petar Bursać, Pranav Minasandra, and Francesca Cagnacci

### Table S1. Mortality data and cause for individuals included in the movement study at the treatment site in Staten Island and at the control site at Rockefeller State Park Preserve in both study years (2021–2022; 2022–2023).

|  | Year 1 | | Year 2 | |
| --- | --- | --- | --- | --- |
|  | SI | RSPP | SI | RSPP |
| Females | 1 DVC (11/20/2021) | 3 Unknown (1/8/2022, 1/30/2022, 3/1/2022)  2 DVC (11/14/2021, 5/29/2021) | 1 Unknown (11/14/2022) | 1 Poaching (12/30/2022) |
| Males | 1 DVC (4/22/2022) | 1 DVC (11/9/2021) | 2 DVC (2/7/23, 3/16/2023) | 1 Poaching (10/29/2022) |

**Table S2.** Individual deer carcasses (monthly percentage of total) that were reported to the New York Department of Sanitation for pick-up on both public and private lands in Staten Island, NY, USA, between July 2016 and June 2024 [1].

|  | 2016–17 | 2017–18 | 2018–19 | 2019–20 | 2020–21 | 2021–22* | 2022–23** | 2023–24 |
| --- | --- | --- | --- | --- | --- | --- | --- | --- |
| July | 8 (5%) | 7 (3%) | 4 (1%) | 8 (6%) | 3 (2%) | 2 (2%) | 3 (5%) | 1 (2%) |
| August | 10 (6%) | 9 (3%) | 8 (3%) | 5 (4%) | 4 (3%) | 8 (9%) | 2 (3%) | 0 (0%) |
| September | 10 (6%) | 14 (5%) | 11 (4%) | 10 (7%) | 7 (5%) | 15 (16%) | 4 (6%) | 2 (4%) |
| October | 22 (13%) | 36 (13%) | 37 (14%) | 19 (13%) | 12 (8%) | 8 (9%) | 13 (20%) | 6 (12%) |
| November | 41 (24%) | 44 (16%) | 52 (19%) | 31 (22%) | 23 (15%) | 6 (7%) | 9 (14%) | 17 (34%) |
| December | 9 (5%) | 21 (8%) | 38 (14%) | 10 (7%) | 30 (20%) | 8 (9%) | 9 (14%) | 5 (10%) |
| January | 13 (8%) | 38 (14%) | 27 (10%) | 17 (12%) | 20 (13%) | 6 (7%) | 5 (8%) | 3 6%) |
| February | 15 (9%) | 30 (11%) | 23 (9%) | 9 (6%) | 12 (8%) | 10 (11%) | 4 (6%) | 4 (8%) |
| March | 10 (6%) | 23 (8%) | 34 (13%) | 6 (4%) | 23 (15%) | 11 (12%) | 3 (5%) | 4 (8%) |
| April | 8 (5%) | 23 (8%) | 13 (5%) | 5 (4%) | 7 (5%) | 5 (5%) | 5 (8%) | 3 (6%) |
| May | 12 (7%) | 24 (9%) | 14 (5%) | 13 (9%) | 4 (3%) | 8 (9%) | 5 (8%) | 3 (6%) |
| June | 11 (7%) | 11 (4%) | 7 (3%) | 9 (6%) | 7 (5%) | 5 (5%) | 3 (5%) | 2 (4%) |
| Total | 169 | 280 | 268 | 142 | 152 | 92 | 65 | 50 |

* Data represent Year 1 of the research program, which was the year an epizootic hemorrhagic disease outbreak occurred.

**Data represent Year 2 of the research program.

References

1. New York City Department of Parks and Recreation (NYCDPR). Managing deer impacts on Staten Island; 2024. https://storymaps.arcgis.com/stories/e3a5f6d544594690a313693d1e88d9ef Accessed 12 Sep 2024
