# Supplementary material for: Effects of vasectomy on breeding-related movement and activity in free-ranging white-tailed deer
Source: Mov Ecol. 2025 May 14;13:34. doi: 10.1186/s40462-025-00554-5 (PMC12079978; doi:10.1186/s40462-025-00554-5)
Supplement: Supplementary file 4 — Additional file 4: Tables S1, S2, and S3: Percentage of movement parameter estimates used in the analysis after excluding NA, non-finite, and excessively large value, families of distributions; percentages of deviance explained, and scale estimates ($\:\widehat{\theta\:}$) for HGAMs; and model summaries for hierarchical generalized additive models for location and scale (HGAMLSs) and hierarchical generalized additive models (HGAMs) [file 40462_2025_554_MOESM4_ESM.docx]

# Additional file 4

**Effects of vasectomy on breeding-related movement and activity in free-ranging white-tailed deer**

Vickie DeNicola, Stefano Mezzini, Petar Bursać, Pranav Minasandra, and Francesca Cagnacci

### Table S1. Percentage of movement parameter estimates used in the analysis after excluding NA, non-finite, and excessively large values, as described in the text.

| Sex (Site) | 7-day home range size (%) | Daily distance traveled (%) | Daily diffusion (%) | Daily excursivity (%) |
| --- | --- | --- | --- | --- |
| Female (RSPP) | 100 | 54.4 | 99.7 | 100 |
| Female (SI) | 97.7 | 55.4 | 99.7 | 100 |
| Male (RSPP) | 99.9 | 75.2 | 96.6 | 100 |
| Male (SI) | 98.7 | 56.2 | 99.7 | 100 |
| Total | 98.9 | 60.3 | 98.9 | 100 |

### Table S2. Families of distributions, percentages of deviance explained, and scale estimates ($\hat{\boldsymbol{\theta}}$) for HGAMs presented, listed by response variable: home range size, daily distance traveled, daily diffusion, daily excursivity, proportion of time spent in no- or low-activity state by day, and number of transitions between states by day. The deviance explained (%) is a measure of goodness of fit that shows the proportion of the total deviance that the model accounts for. Deviance explained is not currently available for the betals family, as it is currently not included in the mgcv package. The scale estimate measures the dispersion of the response variable. Scale estimates are not included for location-scale models because they depend on the covariates. The estimated variance for the Beta and Negative Binomial families, respectively, are $\hat{\boldsymbol{Var(Y)}}\boldsymbol{=}\hat{\boldsymbol{\mu}}\boldsymbol{(1-}\hat{\boldsymbol{\mu}}\boldsymbol{)/(1+}\hat{\boldsymbol{\theta}}\boldsymbol{)}$ and $\hat{\boldsymbol{Var(Y)}}\boldsymbol{=}\hat{\boldsymbol{\mu}}\boldsymbol{+}{\hat{\boldsymbol{\mu}}}^{\boldsymbol{2}}\boldsymbol{/}\hat{\boldsymbol{\theta}}$, where $\hat{\boldsymbol{\mu}}$ is the estimated mean.

| Response variable | Family of distributions | Deviance explained (%) | Scale estimate ($\hat{\theta}$) |
| --- | --- | --- | --- |
| 7-day home range size (km^2^) | Gamma location-scale | 94.5 | Not a constant |
| Daily distance traveled (km/day) | Gamma location-scale | 79.5 | Not a constant |
| Daily diffusion (km^2^/day) | Gamma location-scale | 89.1 | Not a constant |
| Daily excursivity | Beta location-scale | NA | Not a constant |
| Daily proportion of time in no- or low-activity state | Beta | 64.5 | 102.019 |
| Daily number of transitions between states | Negative binomial | 70.7 | 63.215 |

**Table S3.** Model summaries for hierarchical generalized additive models for location and scale (HGAMLSs) and hierarchical generalized additive models (HGAMs) used to estimate the effect of the vasectomy treatment on movement behavior. The response variables are 7-day 95% home range size (km^2^; hr_est_95), daily distance traveled (km/day; speed_est), daily diffusion (km^2^/day; diffusion_est), daily excursivity (unitless; excursivity), the daily proportion of time spent in a no- or low-activity state (vs a medium- or high-activity state; p_low), and the daily number of transitions between activity states (no, low, medium, high; n_transitions). The predictor variables are the four combinations of sex and treatment status (sex_treatment), day of year (days_since_aug_1), study year (study_year), and animal ID for each study year (i.e., each collaring event; animal_year).

| Response variable | Model summary |
| --- | --- |
| 7-day 95% home range size (km^2^; hr_est_95) | Family: gammals  Link function: identity log  Formula:  hr_est_95 ~ sex_treatment + s(days_since_aug_1, by = sex_treatment,  k = 15, bs = "tp") + s(days_since_aug_1, study_year, by = sex_treatment,  k = 15, bs = "sz") + s(days_since_aug_1, animal_year, k = 15,  bs = "fs", xt = list(bs = "cr"))  ~sex_treatment + s(days_since_aug_1, by = sex_treatment, k = 15,  bs = "tp") + s(days_since_aug_1, study_year, by = sex_treatment,  k = 15, bs = "sz") + s(days_since_aug_1, animal_year, k = 15,  bs = "fs", xt = list(bs = "cr"))  Parametric coefficients:  Estimate Std. Error z value Pr(>\|z\|)  (Intercept) -0.83072 0.07205 -11.529 < 2e-16 ***  sex_treatmentf staten_island 0.20332 0.10129 2.007 0.0447 *  sex_treatmentm rockefeller 0.91205 0.13196 6.911 4.80e-12 ***  sex_treatmentm staten_island 0.61185 0.12702 4.817 1.46e-06 ***  (Intercept).1 3.46953 0.09291 37.341 < 2e-16 ***  sex_treatmentf staten_island.1 1.40047 0.13128 10.668 < 2e-16 ***  sex_treatmentm rockefeller.1 1.08324 0.17177 6.306 2.86e-10 ***  sex_treatmentm staten_island.1 1.67883 0.16271 10.318 < 2e-16 ***  ---  Signif. codes: 0 ‘***’ 0.001 ‘**’ 0.01 ‘*’ 0.05 ‘.’ 0.1 ‘ ’ 1  Approximate significance of smooth terms:  edf Ref.df Chi.sq p-value  s(days_since_aug_1):sex_treatmentf rockefeller 11.463 12.027 56.477 < 2e-16 ***  s(days_since_aug_1):sex_treatmentf staten_island 12.652 13.187 102.613 < 2e-16 ***  s(days_since_aug_1):sex_treatmentm rockefeller 11.841 12.487 56.323 2.66e-06 ***  s(days_since_aug_1):sex_treatmentm staten_island 4.759 5.455 3.971 0.60464  s(days_since_aug_1,study_year):sex_treatmentf rockefeller 2.000 2.000 0.757 0.68501  s(days_since_aug_1,study_year):sex_treatmentf staten_island 2.001 2.001 12.864 0.00161 **  s(days_since_aug_1,study_year):sex_treatmentm rockefeller 9.105 9.979 10.837 0.35272  s(days_since_aug_1,study_year):sex_treatmentm staten_island 11.444 12.437 21.654 0.03148 *  s(days_since_aug_1,animal_year) 1000.112 1687.000 72037.164 < 2e-16 ***  s.1(days_since_aug_1):sex_treatmentf rockefeller 11.871 12.908 91.294 < 2e-16 ***  s.1(days_since_aug_1):sex_treatmentf staten_island 11.425 12.485 121.693 < 2e-16 ***  s.1(days_since_aug_1):sex_treatmentm rockefeller 3.996 4.660 4.602 0.40296  s.1(days_since_aug_1):sex_treatmentm staten_island 1.000 1.000 8.409 0.00373 **  s.1(days_since_aug_1,study_year):sex_treatmentf rockefeller 2.000 2.000 5.035 0.08068 .  s.1(days_since_aug_1,study_year):sex_treatmentf staten_island 11.873 13.030 59.071 < 2e-16 ***  s.1(days_since_aug_1,study_year):sex_treatmentm rockefeller 8.831 10.054 17.181 0.07154 .  s.1(days_since_aug_1,study_year):sex_treatmentm staten_island 7.451 8.607 19.899 0.04369 *  s.1(days_since_aug_1,animal_year) 596.901 1670.000 4657.330 < 2e-16 ***  ---  Signif. codes: 0 ‘***’ 0.001 ‘**’ 0.01 ‘*’ 0.05 ‘.’ 0.1 ‘ ’ 1  Deviance explained = 94.5%  -REML = -66.723 Scale est. = 1 n = 9288 |
| Daily distance traveled (km/day; speed_est) | Family: gammals  Link function: identity log  Formula:  speed_est ~ sex_treatment + s(days_since_aug_1, by = sex_treatment,  k = 15, bs = "tp") + s(days_since_aug_1, study_year, by = sex_treatment,  k = 15, bs = "sz") + s(days_since_aug_1, animal_year, k = 15,  bs = "fs", xt = list(bs = "cr"))  ~sex_treatment + s(days_since_aug_1, by = sex_treatment, k = 15,  bs = "tp") + s(days_since_aug_1, study_year, by = sex_treatment,  k = 15, bs = "sz") + s(days_since_aug_1, animal_year, k = 15,  bs = "fs", xt = list(bs = "cr"))  Parametric coefficients:  Estimate Std. Error z value Pr(>\|z\|)  (Intercept) 1.50542 0.03605 41.755 < 2e-16 ***  sex_treatmentf staten_island 0.16129 0.05058 3.189 0.00143 **  sex_treatmentm rockefeller 0.05578 0.06442 0.866 0.38656  sex_treatmentm staten_island 0.10141 0.06345 1.598 0.10998  (Intercept).1 2.78682 0.07110 39.194 < 2e-16 ***  sex_treatmentf staten_island.1 0.60058 0.09920 6.054 1.41e-09 ***  sex_treatmentm rockefeller.1 0.34508 0.12422 2.778 0.00547 **  sex_treatmentm staten_island.1 0.68142 0.12306 5.537 3.07e-08 ***  ---  Signif. codes: 0 ‘***’ 0.001 ‘**’ 0.01 ‘*’ 0.05 ‘.’ 0.1 ‘ ’ 1  Approximate significance of smooth terms:  edf Ref.df Chi.sq p-value  s(days_since_aug_1):sex_treatmentf rockefeller 10.705 12.014 58.004 < 2e-16 ***  s(days_since_aug_1):sex_treatmentf staten_island 11.012 12.260 76.175 < 2e-16 ***  s(days_since_aug_1):sex_treatmentm rockefeller 13.056 13.665 141.405 < 2e-16 ***  s(days_since_aug_1):sex_treatmentm staten_island 12.140 13.189 100.907 < 2e-16 ***  s(days_since_aug_1,study_year):sex_treatmentf rockefeller 6.418 7.442 10.509 0.15144  s(days_since_aug_1,study_year):sex_treatmentf staten_island 11.704 13.011 55.410 2.32e-06 ***  s(days_since_aug_1,study_year):sex_treatmentm rockefeller 2.015 2.019 3.054 0.21978  s(days_since_aug_1,study_year):sex_treatmentm staten_island 2.013 2.016 6.946 0.03111 *  s(days_since_aug_1,animal_year) 538.894 1607.000 15610.431 < 2e-16 ***  s.1(days_since_aug_1):sex_treatmentf rockefeller 1.003 1.004 0.483 0.48469  s.1(days_since_aug_1):sex_treatmentf staten_island 3.242 3.899 14.126 0.00759 **  s.1(days_since_aug_1):sex_treatmentm rockefeller 9.039 10.546 52.670 2.33e-06 ***  s.1(days_since_aug_1):sex_treatmentm staten_island 4.831 5.803 51.349 < 2e-16 ***  s.1(days_since_aug_1,study_year):sex_treatmentf rockefeller 2.670 2.934 1.590 0.57919  s.1(days_since_aug_1,study_year):sex_treatmentf staten_island 2.002 2.002 1.878 0.39124  s.1(days_since_aug_1,study_year):sex_treatmentm rockefeller 2.004 2.006 3.733 0.15448  s.1(days_since_aug_1,study_year):sex_treatmentm staten_island 2.002 2.004 2.869 0.23849  s.1(days_since_aug_1,animal_year) 208.192 1601.000 725.253 < 2e-16 ***  ---  Signif. codes: 0 ‘***’ 0.001 ‘**’ 0.01 ‘*’ 0.05 ‘.’ 0.1 ‘ ’ 1  Deviance explained = 79.6%  -REML = 7886.7 Scale est. = 1 n = 5445 |
| Daily diffusion (km^2^/day; diffusion_est) | Family: gammals  Link function: identity log  Formula:  diffusion_est ~ sex_treatment + s(days_since_aug_1, by = sex_treatment,  k = 15, bs = "tp") + s(days_since_aug_1, study_year, by = sex_treatment,  k = 15, bs = "sz") + s(days_since_aug_1, animal_year, k = 15,  bs = "fs", xt = list(bs = "cr"))  ~sex_treatment + s(days_since_aug_1, by = sex_treatment, k = 15,  bs = "tp") + s(days_since_aug_1, study_year, by = sex_treatment,  k = 15, bs = "sz") + s(days_since_aug_1, animal_year, k = 15,  bs = "fs", xt = list(bs = "cr"))  Parametric coefficients:  Estimate Std. Error z value Pr(>\|z\|)  (Intercept) -3.91469 0.06725 -58.207 < 2e-16 ***  sex_treatmentf staten_island 0.28460 0.09438 3.015 0.00257 **  sex_treatmentm rockefeller 0.53841 0.12211 4.409 1.04e-05 ***  sex_treatmentm staten_island 0.06146 0.11768 0.522 0.60147  (Intercept).1 3.72217 0.06787 54.844 < 2e-16 ***  sex_treatmentf staten_island.1 0.94987 0.09616 9.878 < 2e-16 ***  sex_treatmentm rockefeller.1 0.30712 0.12857 2.389 0.01690 *  sex_treatmentm staten_island.1 0.98776 0.11949 8.267 < 2e-16 ***  ---  Signif. codes: 0 ‘***’ 0.001 ‘**’ 0.01 ‘*’ 0.05 ‘.’ 0.1 ‘ ’ 1  Approximate significance of smooth terms:  edf Ref.df Chi.sq p-value  s(days_since_aug_1):sex_treatmentf rockefeller 11.935 12.545 59.229 2.34e-06 ***  s(days_since_aug_1):sex_treatmentf staten_island 12.794 13.307 171.159 < 2e-16 ***  s(days_since_aug_1):sex_treatmentm rockefeller 13.073 13.453 143.199 < 2e-16 ***  s(days_since_aug_1):sex_treatmentm staten_island 11.843 12.661 64.331 < 2e-16 ***  s(days_since_aug_1,study_year):sex_treatmentf rockefeller 2.014 2.017 1.375 0.505948  s(days_since_aug_1,study_year):sex_treatmentf staten_island 10.467 11.540 14.520 0.263509  s(days_since_aug_1,study_year):sex_treatmentm rockefeller 8.586 9.507 14.627 0.105467  s(days_since_aug_1,study_year):sex_treatmentm staten_island 2.046 2.056 7.894 0.017046 *  s(days_since_aug_1,animal_year) 957.768 1679.000 53860.569 < 2e-16 ***  s.1(days_since_aug_1):sex_treatmentf rockefeller 9.313 10.713 37.534 9.01e-05 ***  s.1(days_since_aug_1):sex_treatmentf staten_island 10.372 11.638 110.147 < 2e-16 ***  s.1(days_since_aug_1):sex_treatmentm rockefeller 7.576 8.862 32.581 0.000227 ***  s.1(days_since_aug_1):sex_treatmentm staten_island 5.272 6.264 25.602 0.000359 ***  s.1(days_since_aug_1,study_year):sex_treatmentf rockefeller 2.013 2.017 2.402 0.302130  s.1(days_since_aug_1,study_year):sex_treatmentf staten_island 11.696 12.944 70.499 < 2e-16 ***  s.1(days_since_aug_1,study_year):sex_treatmentm rockefeller 10.025 11.417 45.790 1.43e-05 ***  s.1(days_since_aug_1,study_year):sex_treatmentm staten_island 2.011 2.014 2.461 0.294433  s.1(days_since_aug_1,animal_year) 469.380 1667.000 2699.844 < 2e-16 ***  ---  Signif. codes: 0 ‘***’ 0.001 ‘**’ 0.01 ‘*’ 0.05 ‘.’ 0.1 ‘ ’ 1  Deviance explained = 89.1%  -REML = -30616 Scale est. = 1 n = 9320 |
| Daily excursivity (unitless; excursivity) | Family: betals  Link function: identity identity  Formula:  density ~ sex_treatment + s(days_since_aug_1, by = sex_treatment,  k = 15, bs = "tp") + s(days_since_aug_1, study_year, by = sex_treatment,  k = 15, bs = "sz") + s(days_since_aug_1, animal_year, k = 15,  bs = "fs", xt = list(bs = "cr"))  ~sex_treatment + s(days_since_aug_1, by = sex_treatment, k = 15,  bs = "tp") + s(days_since_aug_1, study_year, by = sex_treatment,  k = 15, bs = "sz") + s(days_since_aug_1, animal_year, k = 15,  bs = "fs", xt = list(bs = "cr"))  Parametric coefficients:  Estimate Std. Error z value Pr(>\|z\|)  (Intercept) -0.19699 0.05235 -3.763 0.000168 ***  sex_treatmentf staten_island -0.29277 0.07335 -3.992 6.56e-05 ***  sex_treatmentm rockefeller -0.15977 0.09633 -1.658 0.097217 .  sex_treatmentm staten_island -0.23254 0.09171 -2.536 0.011227 *  (Intercept).1 -2.69152 0.05645 -47.680 < 2e-16 ***  sex_treatmentf staten_island.1 0.06305 0.07914 0.797 0.425664  sex_treatmentm rockefeller.1 0.37802 0.10364 3.647 0.000265 ***  sex_treatmentm staten_island.1 0.33703 0.09939 3.391 0.000697 ***  ---  Signif. codes: 0 ‘***’ 0.001 ‘**’ 0.01 ‘*’ 0.05 ‘.’ 0.1 ‘ ’ 1  Approximate significance of smooth terms:  edf Ref.df Chi.sq p-value  s(days_since_aug_1):sex_treatmentf rockefeller 11.786 12.562 61.758 < 2e-16 ***  s(days_since_aug_1):sex_treatmentf staten_island 11.898 12.699 51.020 5.09e-06 ***  s(days_since_aug_1):sex_treatmentm rockefeller 13.142 13.579 195.427 < 2e-16 ***  s(days_since_aug_1):sex_treatmentm staten_island 7.681 8.807 34.464 7.87e-05 ***  s(days_since_aug_1,study_year):sex_treatmentf rockefeller 2.003 2.004 0.937 0.626764  s(days_since_aug_1,study_year):sex_treatmentf staten_island 2.041 2.050 4.853 0.082115 .  s(days_since_aug_1,study_year):sex_treatmentm rockefeller 11.374 12.460 24.608 0.013818 *  s(days_since_aug_1,study_year):sex_treatmentm staten_island 7.049 8.001 3.853 0.869719  s(days_since_aug_1,animal_year) 969.229 1696.000 55845.107 < 2e-16 ***  s.1(days_since_aug_1):sex_treatmentf rockefeller 9.982 11.333 19.673 0.055103 .  s.1(days_since_aug_1):sex_treatmentf staten_island 10.884 12.123 50.849 6.03e-06 ***  s.1(days_since_aug_1):sex_treatmentm rockefeller 1.014 1.018 0.042 0.856879  s.1(days_since_aug_1):sex_treatmentm staten_island 1.005 1.006 0.131 0.720167  s.1(days_since_aug_1,study_year):sex_treatmentf rockefeller 2.005 2.006 1.028 0.599549  s.1(days_since_aug_1,study_year):sex_treatmentf staten_island 8.070 9.271 7.745 0.481704  s.1(days_since_aug_1,study_year):sex_treatmentm rockefeller 2.014 2.017 1.513 0.473243  s.1(days_since_aug_1,study_year):sex_treatmentm staten_island 11.262 12.593 35.179 0.000566 ***  s.1(days_since_aug_1,animal_year) 790.163 1681.000 6465.661 < 2e-16 ***  ---  Signif. codes: 0 ‘***’ 0.001 ‘**’ 0.01 ‘*’ 0.05 ‘.’ 0.1 ‘ ’ 1  Deviance explained = NA%  -REML = -16743 Scale est. = 1 n = 29856 |
| Daily proportion of time spent in a no- or low-activity state (vs a medium- or high-activity state; p_low) | Family: Beta regression(102.019)  Link function: logit  Formula:  p_low ~ sex_treatment + s(days_since_aug_1, by = sex_treatment,  k = 10, bs = "tp") + s(days_since_aug_1, study_year, by = sex_treatment,  k = 10, bs = "sz") + s(days_since_aug_1, animal_year, k = 10,  bs = "fs", xt = list(bs = "cr"))  Parametric coefficients:  Estimate Std. Error t value Pr(>\|t\|)  (Intercept) 0.13369 0.02281 5.860 4.68e-09 ***  sex_treatmentf staten_island -0.03043 0.03204 -0.950 0.342  sex_treatmentm rockefeller -0.01673 0.04714 -0.355 0.723  sex_treatmentm staten_island 0.19752 0.03987 4.954 7.30e-07 ***  ---  Signif. codes: 0 ‘***’ 0.001 ‘**’ 0.01 ‘*’ 0.05 ‘.’ 0.1 ‘ ’ 1  Approximate significance of smooth terms:  edf Ref.df F p-value  s(days_since_aug_1):sex_treatmentf rockefeller 8.781 8.843 21.299 < 2e-16 ***  s(days_since_aug_1):sex_treatmentf staten_island 8.338 8.531 12.249 < 2e-16 ***  s(days_since_aug_1):sex_treatmentm rockefeller 6.286 6.670 2.873 0.00436 **  s(days_since_aug_1):sex_treatmentm staten_island 8.371 8.517 7.497 < 2e-16 ***  s(days_since_aug_1,study_year):sex_treatmentf rockefeller 2.013 2.016 0.137 0.87511  s(days_since_aug_1,study_year):sex_treatmentf staten_island 9.269 9.479 5.687 < 2e-16 ***  s(days_since_aug_1,study_year):sex_treatmentm rockefeller 2.048 2.056 0.128 0.88945  s(days_since_aug_1,study_year):sex_treatmentm staten_island 6.133 6.570 1.196 0.30591  s(days_since_aug_1,animal_year) 820.849 1069.000 25.378 < 2e-16 ***  ---  Signif. codes: 0 ‘***’ 0.001 ‘**’ 0.01 ‘*’ 0.05 ‘.’ 0.1 ‘ ’ 1  R-sq.(adj) = 0.621 Deviance explained = 64.4%  fREML = -17043 Scale est. = 1 n = 28168 |
| Daily number of transitions between activity states (no, low, medium, high; n_transitions) | Family: Negative Binomial(63.215)  Link function: log  Formula:  n_transitions ~ sex_treatment + s(days_since_aug_1, by = sex_treatment,  k = 10, bs = "tp") + s(days_since_aug_1, study_year, by = sex_treatment,  k = 10, bs = "sz") + s(days_since_aug_1, animal_year, k = 10,  bs = "fs", xt = list(bs = "cr"))  Parametric coefficients:  Estimate Std. Error t value Pr(>\|t\|)  (Intercept) 8.123078 0.017926 453.134 <2e-16 ***  sex_treatmentf staten_island 0.022158 0.024967 0.887 0.375  sex_treatmentm rockefeller 0.043288 0.036710 1.179 0.238  sex_treatmentm staten_island 0.005262 0.030953 0.170 0.865  ---  Signif. codes: 0 ‘***’ 0.001 ‘**’ 0.01 ‘*’ 0.05 ‘.’ 0.1 ‘ ’ 1  Approximate significance of smooth terms:  edf Ref.df F p-value  s(days_since_aug_1):sex_treatmentf rockefeller 8.681 8.784 18.200 < 2e-16 ***  s(days_since_aug_1):sex_treatmentf staten_island 8.528 8.675 18.164 < 2e-16 ***  s(days_since_aug_1):sex_treatmentm rockefeller 8.816 8.876 29.269 < 2e-16 ***  s(days_since_aug_1):sex_treatmentm staten_island 8.769 8.833 18.137 < 2e-16 ***  s(days_since_aug_1,study_year):sex_treatmentf rockefeller 8.488 8.858 2.895 0.00316 **  s(days_since_aug_1,study_year):sex_treatmentf staten_island 9.243 9.467 6.391 < 2e-16 ***  s(days_since_aug_1,study_year):sex_treatmentm rockefeller 6.641 7.098 1.008 0.54605  s(days_since_aug_1,study_year):sex_treatmentm staten_island 7.574 8.018 3.076 0.00180 **  s(days_since_aug_1,animal_year) 808.212 1071.000 41.796 < 2e-16 ***  ---  Signif. codes: 0 ‘***’ 0.001 ‘**’ 0.01 ‘*’ 0.05 ‘.’ 0.1 ‘ ’ 1  R-sq.(adj) = 0.694 Deviance explained = 70.7%  fREML = 42373 Scale est. = 1 n = 28220 |
