# Supplementary material for: Effects of vasectomy on breeding-related movement and activity in free-ranging white-tailed deer
Source: Mov Ecol. 2025 May 14;13:34. doi: 10.1186/s40462-025-00554-5 (PMC12079978; doi:10.1186/s40462-025-00554-5)
Supplement: Supplementary file 5 — Additional file 5: Section S1 and Figure S1: Detailed description of data cleaning and processing for telemetry data from both study sites and median GPS fix rate for individuals in the final sample [file 40462_2025_554_MOESM5_ESM.docx]

# Additional file 5

**Effects of vasectomy on breeding-related movement and activity in free-ranging white-tailed deer**

Vickie DeNicola, Stefano Mezzini, Petar Bursać, Pranav Minasandra, and Francesca Cagnacci

**Section S1.** **Detailed description of data cleaning and processing for telemetry data from both study sites.**

After downloading raw GPS data (n = 735,270) from MoveBank, we removed events without coordinates (n = 15,854), those from individuals tracked for < 28 days, and those from individuals in Year 1 who died of unknown causes before the first frost (known epizootic hemorrhagic disease outbreak; n = 20,566). We removed ⁠outlier locations (n = 37) based on the visual inspection of telemetry data and the relationships between the minimum speed (assuming straight-line displacement) and deviation from the median location, turning angle, and time between locations. For each individual, we removed the first 10 and last 2 days of data from the analysis to reduce disturbance from the capture event and to limit the potential for inclusion of data after the collar had dropped off [1]. The median sampling interval for each individual included in the final sample is shown in Fig. S1A.

**References**

1. Morellet N, Verheyden H, Angibault JM, Cargnelutti B, Lourtet B, Hewison MA. The effect of capture on ranging behaviour and activity of the European roe deer Capreolus capreolus. Wildl Biol. 2009;15:278–87. <https://doi.org/10.2981/08-084>

###
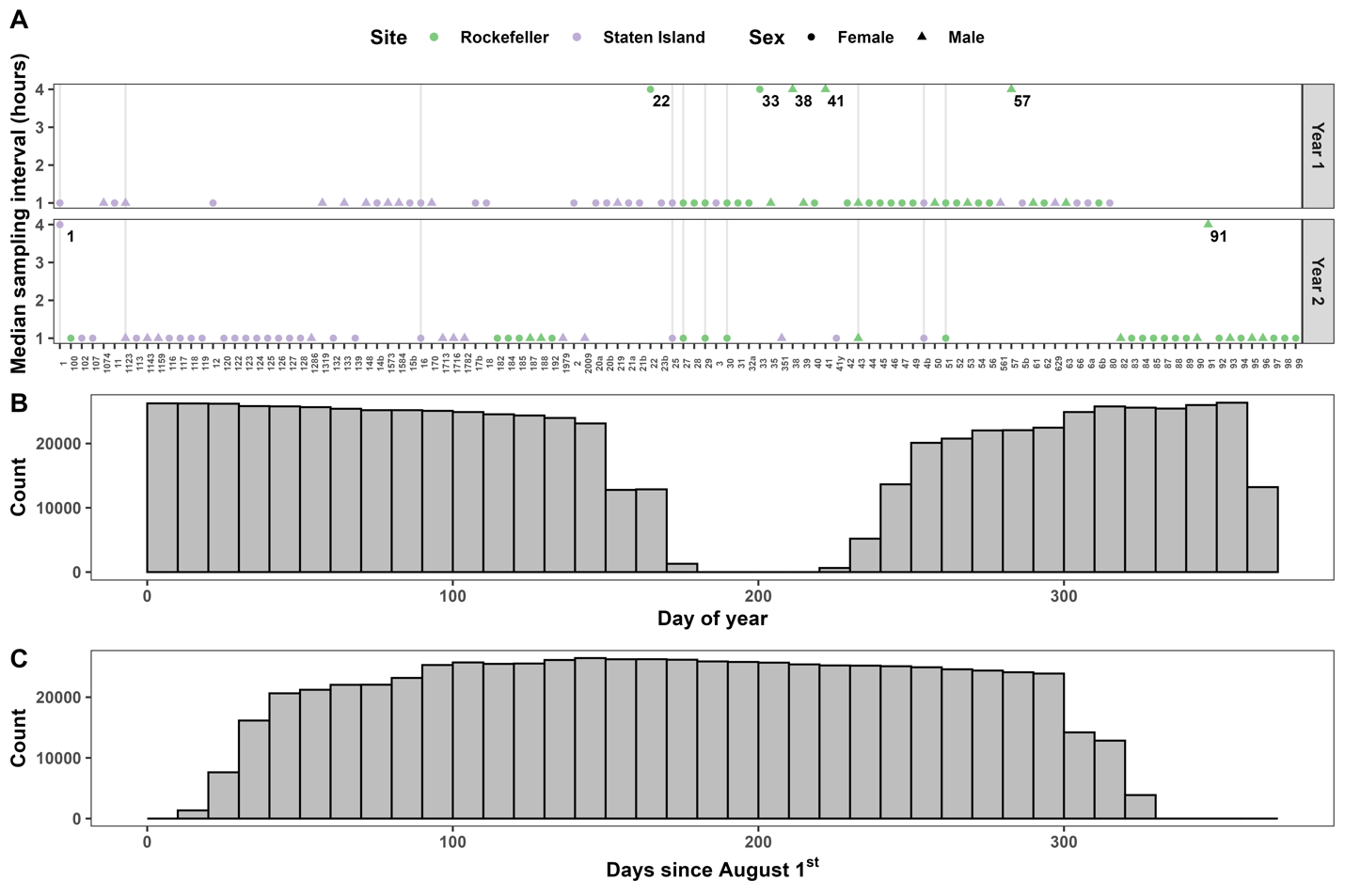


### Figure S1. Median GPS fix rate for individuals in the final sample. Text labels indicate which deer had a 4-h sampling interval, whereas gray vertical lines indicate which deer were tracked in both years (A). Total number of GPS fixes over day of year (i.e., days since December 31^st^ of the previous year; (B) and days since 1 August (C). In panel B, note the lack of continuity between December and January as well as the gap between the end of data collection in May/June and the deployment of collars and contrast this with panel B, where dates are shown as the number of days since 1 August.
