# Supplementary material for: Effects of vasectomy on breeding-related movement and activity in free-ranging white-tailed deer
Source: Mov Ecol. 2025 May 14;13:34. doi: 10.1186/s40462-025-00554-5 (PMC12079978; doi:10.1186/s40462-025-00554-5)
Supplement: Supplementary file 6 — Additional file 6: Figure S1: Telemetry data (dots) and autocorrelated kernel density estimate (shading) for deer 148 in Year 1 (2021–22) with a two-point equidistant projection [file 40462_2025_554_MOESM6_ESM.docx]

# Additional file 6

**Effects of vasectomy on breeding-related movement and activity in free-ranging white-tailed deer**

Vickie DeNicola, Stefano Mezzini, Petar Bursać, Pranav Minasandra, and Francesca Cagnacci


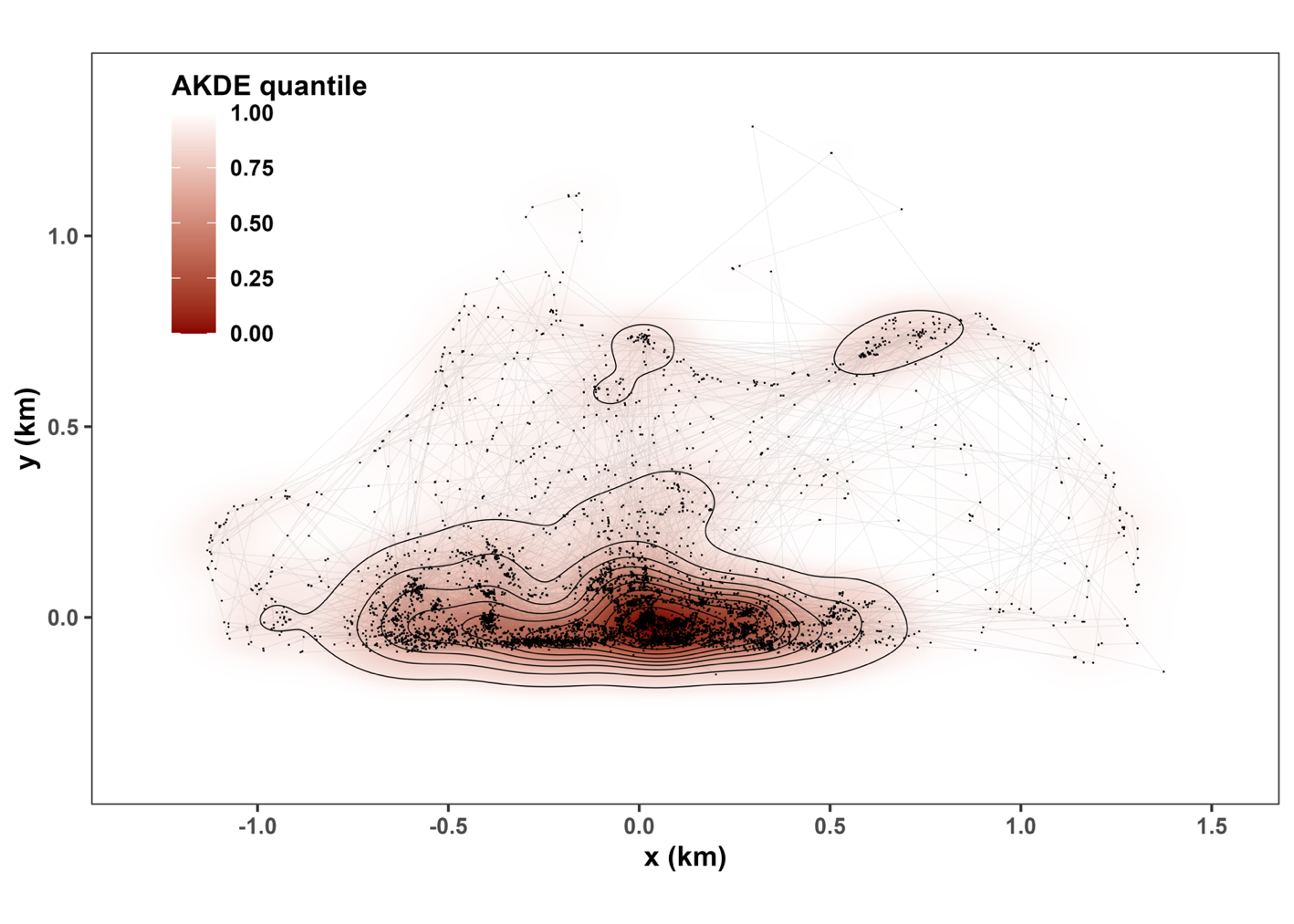


### Figure S1. Telemetry data (dots) and autocorrelated kernel density estimate (shading) for deer 148 in Year 1 (2021–22) with a two-point equidistant projection. The contours indicate quantiles at intervals of 0.1. Excursivity was measured by taking the average quantile of the locations for each day. A value close to 0 indicated the deer used high-use areas, whereas a value of 1 indicated that the deer used more “peripheral” areas that were generally only used during excursions.
