# Supplementary material for: Effects of vasectomy on breeding-related movement and activity in free-ranging white-tailed deer
Source: Mov Ecol. 2025 May 14;13:34. doi: 10.1186/s40462-025-00554-5 (PMC12079978; doi:10.1186/s40462-025-00554-5)
Supplement: Supplementary file 7 — Additional file 7: Figures S1 and S2: Monthly trimodal distribution of log mean VeDBA values for 2-s intervals for one individual and trimodal distribution of log mean VeDBA values for 2-s intervals for one individual. Low (log(VeDBA) < 1.12), medium (1.12 < log(VeDBA) < 4.2), and high (log(VeDBA) > 4.2) activity states are represented by the three distribution peaks [file 40462_2025_554_MOESM7_ESM.docx]

# **Additional file 7**

**Effects of vasectomy on breeding-related movement and activity in free-ranging white-tailed deer**

Vickie DeNicola, Stefano Mezzini, Petar Bursać, Pranav Minasandra, and Francesca Cagnacci

**
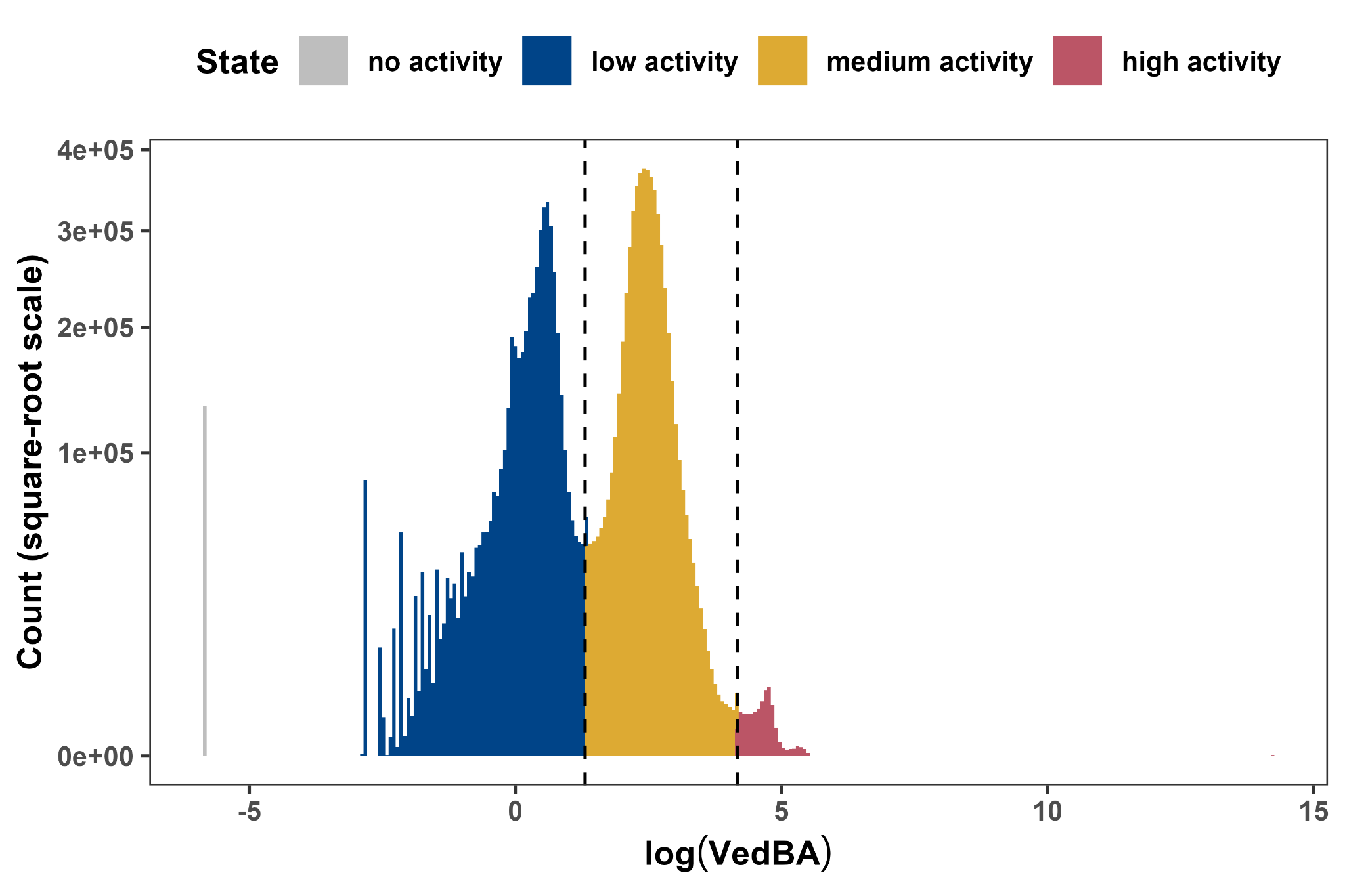
**

### **Figure S1.** Trimodal distribution of log mean VeDBA values for 2-s intervals for one individual. Low (log(VeDBA) < 1.12), medium (1.12 < log(VeDBA) < 4.2), and high (log(VeDBA) > 4.2) activity states are represented by the three distribution peaks. The small peak represents the high activity state at ~4.5. The same 3-peak pattern was observed in all individuals. VeDBA values of 0 (log(VeDBA) = -$\infty$) are shown at the minimum finite log(VeDBA) - 3 for ease of readability.


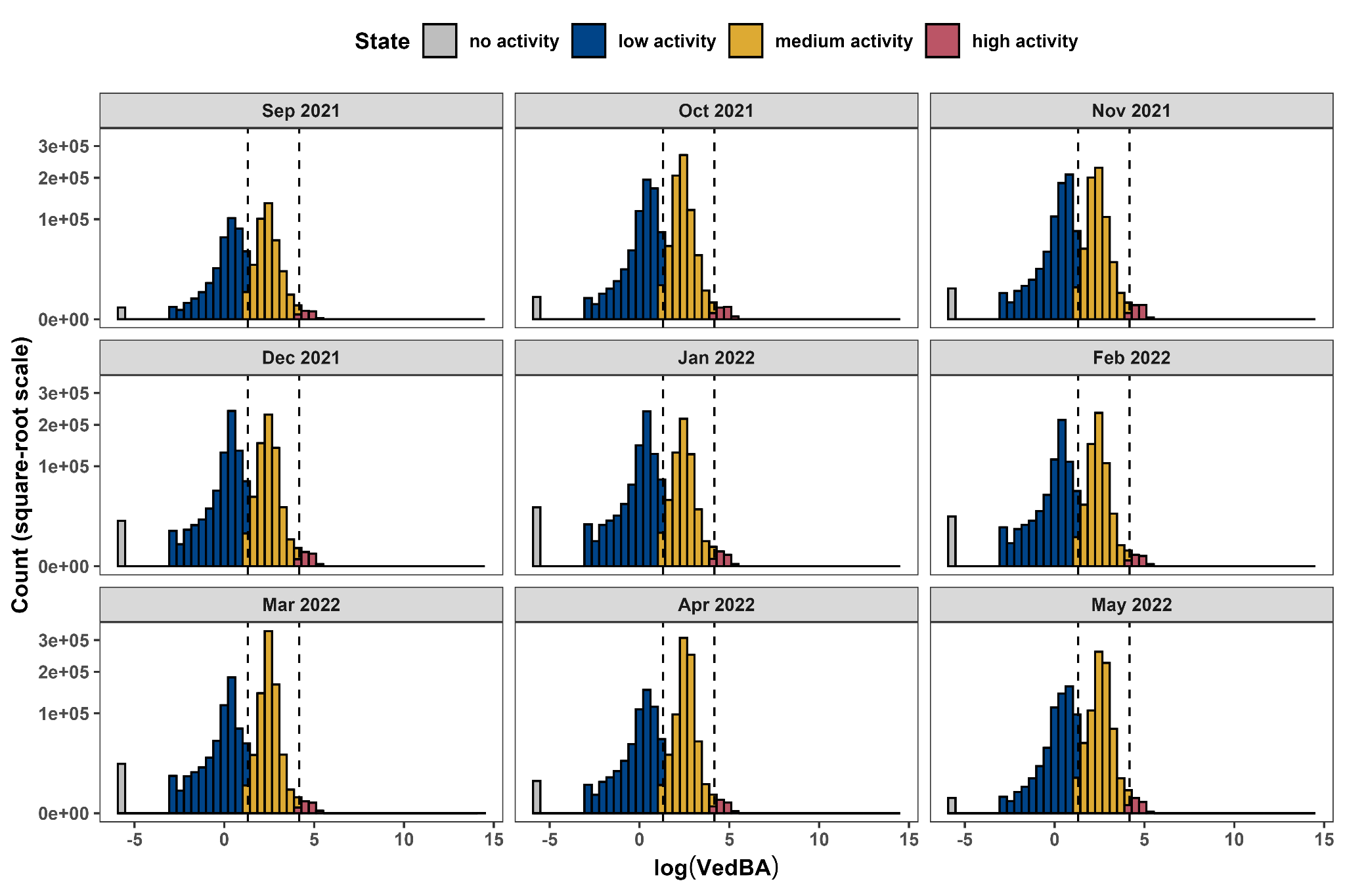
**Figure S2.** Monthly trimodal distribution of log mean VeDBA values for 2-s intervals for one individual. The boundaries of low, medium, and high states did not change by month when 10 individuals were examined.
