# Supplementary material for: Effects of vasectomy on breeding-related movement and activity in free-ranging white-tailed deer
Source: Mov Ecol. 2025 May 14;13:34. doi: 10.1186/s40462-025-00554-5 (PMC12079978; doi:10.1186/s40462-025-00554-5)
Supplement: Supplementary file 8 — Additional file 8: Table S1: Percentage habitat composition for female and male deer at treatment and control sites in Year 1. Habitat composition was determined using GLC_FCS30D land cover data (Liangyun et al. 2023) overlaid with the 50% home range estimated via Autocorrelated Kernel Density Estimation (AKDE) in ctmm (Calabrese et al. 2016). The area of each habitat type within the 50% AKDE was calculated for each individual, and group-level percentages represent the habitat composition across all individuals in that category [file 40462_2025_554_MOESM8_ESM.docx]

# **Additional file 8**

**Effects of vasectomy on breeding-related movement and activity in free-ranging white-tailed deer**

Vickie DeNicola, Stefano Mezzini, Petar Bursać, Pranav Minasandra, and Francesca Cagnacci

**Table S1-4.** Percentage habitat composition for female and male deer at treatment and control sites in Year 1. Habitat composition was determined using GLC_FCS30D land cover data (Liangyun et al. 2023) overlaid with the 50% home range estimated via Autocorrelated Kernel Density Estimation (AKDE) in ctmm (Calabrese et al. 2016). The area of each habitat type within the 50% AKDE was calculated for each individual, and group-level percentages represent the habitat composition across all individuals in that category.

| **Control site females** |  |
| --- | --- |
| **Habitat class** | **%** |
| Closed deciduous broadleaved forest | 77.18 |
| Herbaceous cover cropland | 14.28 |
| Impervious surfaces | 3.07 |
| Shrubland | 1.34 |
| Closed evergreen needle-leaved forest | 1.17 |
| Open evergreen broadleaved forest | 0.96 |
| Open mixed leaf forest (broadleaved and needle-leaved) | 0.57 |
| Grassland | 0.54 |
| Rainfed cropland | 0.43 |
| Open evergreen needle-leaved forest | 0.28 |
| Open deciduous broadleaved forest | 0.12 |
| Irrigated cropland | 0.05 |
|  |  |
|  |  |
| **Control site males** |  |
| **Habitat class** | **%** |
| Closed deciduous broadleaved forest | 70.17 |
| Impervious surfaces | 12.71 |
| Herbaceous cover cropland | 8.39 |
| Closed evergreen needle-leaved forest | 3.58 |
| Rainfed cropland | 1.04 |
| Shrubland | 0.99 |
| Open evergreen needle-leaved forest | 0.7 |
| Open mixed leaf forest (broadleaved and needle-leaved) | 0.53 |
| Water body | 0.5 |
| Open evergreen broadleaved forest | 0.34 |
| Salt marsh | 0.24 |
| Grassland | 0.2 |
| Swamp | 0.2 |
| Open deciduous needle-leaved forest | 0.09 |
| Open deciduous broadleaved forest | 0.07 |
| Closed deciduous needle-leaved forest (fc >0.4) | 0.06 |
| Irrigated cropland | 0.04 |
| Marsh | 0.04 |
| Closed evergreen broadleaved forest | 0.03 |
| Sparse vegetation | 0.03 |
| Bare areas | 0.01 |
| Evergreen shrubland | 0.01 |

| **Treatment site females** |  |
| --- | --- |
| **Habitat class** | **%** |
| Closed deciduous broadleaved forest | 46.61 |
| Impervious surfaces | 24.18 |
| Herbaceous cover cropland | 12.82 |
| Closed evergreen needle-leaved forest | 4.89 |
| Salt marsh | 4.76 |
| Shrubland | 1.88 |
| Open evergreen broadleaved forest | 1.15 |
| Open evergreen needle-leaved forest | 0.85 |
| Rainfed cropland | 0.53 |
| Water body | 0.53 |
| Tidal flat | 0.37 |
| Lichens and mosses | 0.32 |
| Swamp | 0.3 |
| Closed deciduous needle-leaved forest (fc >0.4) | 0.27 |
| Open mixed leaf forest (broadleaved and needle-leaved) | 0.19 |
| Grassland | 0.16 |
| Open deciduous broadleaved forest | 0.09 |
| Sparse vegetation | 0.09 |

| **Treatment site males** |  |
| --- | --- |
| **Habitat class** | **%** |
| Impervious surfaces | 37.71 |
| Closed deciduous broadleaved forest | 24 |
| Herbaceous cover cropland | 21.29 |
| Water body | 4.01 |
| Salt marsh | 3.93 |
| Closed evergreen needle-leaved forest | 3.2 |
| Open evergreen broadleaved forest | 1.29 |
| Rainfed cropland | 1 |
| Swamp | 0.92 |
| Open evergreen needle-leaved forest | 0.81 |
| Shrubland | 0.65 |
| Open deciduous broadleaved forest | 0.34 |
| Marsh | 0.26 |
| Grassland | 0.14 |
| Sparse vegetation | 0.14 |
| Tidal flat | 0.14 |
| Open mixed leaf forest (broadleaved and needle-leaved) | 0.06 |
| Bare areas | 0.03 |
| Closed deciduous needle-leaved forest (fc >0.4) | 0.03 |
| Irrigated cropland | 0.03 |
| Closed evergreen broadleaved forest | 0.01 |

**References**

Calabrese JM, Fleming CH, Gurarie E. ctmm: an R package for analyzing animal relocation data as a continuous‐time stochastic process. Methods Ecol Evol. 2016;7:1124–32. [https://doi.org/10.1111/2041-210X.12559](https://doi.org/10.1111/2041-210x.12559)

Liangyun Liu, Xiao Zhang, & Tingting Zhao. (2023). GLC_FCS30D: the first global 30-m land-cover dynamic monitoring product with fine classification system from 1985 to 2022 [Data set]. Zenodo. <https://doi.org/10.5281/zenodo.8239305>
